# Direct-to-consumer self-tests sold in the UK in 2023: cross sectional review of information on intended use, instructions for use, and post-test decision making

**DOI:** 10.1136/bmj-2025-085546

**Published:** 2025-07-23

**Authors:** Clare Davenport, Alex Richter, Bethany Hillier, Katie Scandrett, Ridhi Agarwal, Simon W Baldwin, Aditya U Kale, Joseph Alderman, Trystan Macdonald, Jonathan J Deeks

**Affiliations:** 1 Department of Applied Health Sciences, School of Health Sciences, College of Medicine and Health, University of Birmingham, Birmingham, UK; 2 NIHR Birmingham Biomedical Research Centre, Birmingham, University Hospitals Birmingham NHS Foundation Trust and University of Birmingham, Birmingham, UK; 3 Department of Clinical Immunology Services, School of Infection, Inflammation and Immunology, College of Medicine and Health, University of Birmingham, Birmingham, UK; 4 University Hospitals Birmingham NHS Foundation Trust, Birmingham, UK; 5 Department of Inflammation and Ageing, School of Infection, Inflammation and Immunology, College of Medicine and Health, University of Birmingham, Birmingham, UK

## Abstract

**Objectives:**

To review the information provided for self-test devices sold in high street shops in the UK and to assess their suitability for informed decision making based on use, interpretation, and post-test actions.

**Design:**

Cross sectional review of information on self-test boxes and instructions for use leaflets.

**Setting:**

Supermarkets, pharmacies, and health and wellbeing shops within a 10 mile radius of the University of Birmingham’s campus at Edgbaston in 2023.

**Main outcome measures:**

Information on intended use of test, biomarker and clinical condition, interpretation of test results, recommendations for post-test actions, and coherence of intended use and post-test recommendations with evidence based guidance.

**Results:**

30 self-tests assessing 20 biomarkers for 19 different conditions were included. Information to guide purchase was present on a few boxes: who should use the test and when (8/30, 27%), action after the test result (7/30, 23%), and numerical test performance (10/30, 33%). From the information provided either on the box or within the instructions for use leaflets, 21 (70%) self-tests were judged to be used for diagnosis and 15 (50%) to be used for screening, although 3/21 (14%) did not provide any information about symptoms and 10/15 (67%) did not provide any information about risk factors to guide use. 27 (90%) self-tests recommended follow-up with a healthcare professional if results were positive or abnormal, and 14 (47%) if test results were negative or normal. Use of tests for 11 of 19 (58%) conditions was judged contrary to evidence based guidance in one or more of the intended population, frequency of testing, test threshold, or investigative approach required for a condition.

**Conclusions:**

The current market for self-tests does not support consumer informed decisions about their use, interpretation of test results, and subsequent actions. Clinicians working downstream of self-tests are likely to face important challenges in incorporating the results in practice. As the use of self-tests continues to increase, improved regulatory oversight is urgently needed to protect the public and healthcare systems from misuse.

## Introduction

In recent years the availability and use of direct-to-consumer tests has expanded substantially in the UK and globally.^1^ The covid-19 pandemic accelerated this trend, with government endorsement and the widespread use of self-testing, alongside global advancements in the manufacturing of lateral flow tests. This increase in self-testing occurs against a backdrop of increasing healthcare demands that exceed NHS capacity.^2^ ^3^ The recent increase in direct-to-consumer tests has the potential to generate inappropriate demand on an already stretched primary care system,^4^ as the costs for follow-up from privately purchased tests are borne by a publicly funded healthcare system.^5^

Direct-to-consumer tests are commercial tests available directly to the public. Different modalities of such tests have been described,^6^ including self-testing (all aspects of the testing process are done by the user: the decision to test, sampling, conduct and interpretation of the test, and post-test decision making), self-sampling (the decision to test, sampling, and post-test decision making are done by the user, but the conduct and interpretation of the test are done by professionals), and direct access testing (the decision to test is initiated by the consumer, but sampling, conduct and interpretation of the test, and post-test decision making are done or supported by healthcare professionals). The key factor that all direct-to-consumer tests share is that the decision to test is not initiated or recommended by a healthcare professional.

Direct-to-consumer tests may be appealing to the public as they can provide diagnostic results quickly, offering privacy, confidentiality, and autonomy over healthcare decisions. When integrated appropriately into clinical pathways, self-tests have been shown to increase uptake of testing in underserved groups, such as those at risk of HIV.^7^ Additionally, direct-to-consumer tests have the potential to improve diagnosis and monitoring amid constraints on NHS resources.^8^ ^9^

However, direct-to-consumer tests also pose risks to individuals and the broader healthcare system. In the absence of guidance from healthcare professionals, individuals might use tests inappropriately or without a clear understanding of the implications of the results. False positive test results can lead to unnecessary anxiety, increased healthcare usage, and additional costs, whereas false negative test results may delay appropriate treatment or engagement with evidence based, government funded screening programmes. Test errors can stem from inherent limitations in the accuracy of the test, as well as user related issues such as sampling errors, incorrect processing, and difficulties in interpreting the results.

We undertook a cross sectional review to assess whether direct-to-consumer self-test devices sold in high street shops in the UK are fit for their specified purposes, can benefit the public and reduce demands on the health service, and are safe and reliable. This report is one of two generated from the study. Our paired paper^10^ assessed claims about performance and the supporting evidence base, readability of the documents, reliability and ergonomics of the equipment, sampling method, and instructions. This report assesses the clarity of information provided to consumers about the intended use of a self-test, instructions for use (IFU), and recommended actions after a result. Specifically, we evaluated the clarity of the intended purpose of testing (whether the test is being used for screening, diagnosis, or monitoring), the population to be tested, the biomarker and clinical condition the test is designed to detect; IFU, including how to interpret test results and recommendations for actions to be taken after a test result (normal or abnormal); and the coherence of intended use and recommendations for actions to be taken after a test result with national or international evidence based guidance.

## Methods

### Sampling

Using a convenience sampling approach, we aimed to comprehensively assess self-tests marketed in high street shops in the UK. We identified self-tests on sale in supermarkets, community pharmacies, and health and wellbeing shops located within a 10 mile (16 km) radius of the University of Birmingham’s campus at Edgbaston. This area encompasses largely urban areas of the metropolitan boroughs of Birmingham, Dudley, Sandwell, Solihull, and Walsall, with a combined population of about 2.6 million, representing 4.4% of the population of England and Wales.^11^ The shopping areas include stores of all the main UK supermarkets, pharmacies, and health and wellbeing shops. High street retailers selling self-tests were identified through an online business directory (yell.com). Only businesses with 10 or more UK outlets were selected as this was considered likely to provide a generalisable snapshot of nationwide availability. We identified self-tests on the websites and shelves of each store, and during a visit purchased a single example of each available test kit. Sampling was conducted in April 2023.

We included self-tests where the sample is intended to be taken, tested, and interpreted by the user. Other direct-to-consumer tests, such as self-sampling tests, where samples are taken by the user but sent to a laboratory for processing and interpretation, were excluded. We also excluded pregnancy tests and ovulation tests as these are already established in community use; self-tests for detecting alcohol or drug misuse as they do not have the intended purpose of detecting or monitoring a disease or health condition; and test strips used as part of a test meter (as with a blood glucose monitor).

### Information sources and data extraction

For each test, we extracted information from the box and from the IFU leaflet, which is sometimes labelled as the patient information sheet. This information was extracted by one researcher and checked by a second researcher, and any discrepancies were resolved through discussion with additional members of the project team as necessary. Extraction of information was performed verbatim. When judgments were made in the absence of explicit information, we clearly indicate this in the results as being “implicit.” For example, we would make an implicit judgment that the test application was for diagnosis if symptoms were provided as an indication for test use. We identified and tabulated data, including characteristics of the device (biomarker, sample type, cost), statements on intended use (purpose, population, clinical condition), instructions for interpreting the results and recommended actions after a test result, and coherence of intended use and post-test recommended actions with national and international guidelines.

All co-investigators, including test experts, statisticians, clinicians, and a test manufacturer, met to review and evaluate the test, sampling equipment, and IFU leaflet. This paper reports their assessments based on information about intended use of the self-test, IFU, interpretation of test results, and recommended post-test actions.

To identify evidence based practice relevant to the selected tests, we included guidance for the intended clinical condition (and in the absence of a clear clinical condition, for the biomarker mentioned). Guidance was identified from the National Institute for Health and Care Excellence (NICE), National Screening Committee, and World Health Organization (WHO). Guidance for use of the test (laboratory, point-of-care, or self-test version) was compared with information provided by the tests in our sample for intended purpose of testing (screening, diagnosis, monitoring), indications for testing (age, biological sex, presence of symptoms), thresholds used within healthcare systems in the UK (when applicable), place of the test in a testing strategy (for example, should the test be used as an initial investigation, should the test be used alone or in combination with other tests), and recommended frequency of testing (when applicable). Based on this information we judged whether information provided for the self-tests was coherent with evidence based guidance.

### Patient and public involvement

Public engagement was conducted at a research showcase event at Queen Elizabeth Hospital, Birmingham, on 19 May 2023 where attendees were shown posters and packaging of self-tests we had purchased. Twenty members of the public and 30 healthcare professionals engaged and were asked about their awareness and experiences of self-tests, trust in the results, likelihood of using the self-tests, and the type of information they considered important to know about a test (supplementary table A1 lists the questions asked). Insights from this exercise informed the data extraction framework.

## Results

### Test characteristics

We identified 35 self-tests sold by outlets within the 10 mile radius of the University of Birmingham’s campus at Edgbaston (table 1). Five tests were excluded: three were clones of other tests already in the sample (differing only in name of the distributors) and two were out of stock at the time of sampling (Cholesterol Home Test Kit, Boots Pharmaceuticals; HIV Self-Test Oraquick, Superdrug). Table 1 provides information on each of the 30 included tests, labelled with identifiers T1-T30. Three tests (T3, T26, and T28) were close clones of other tests (T4, T25, and T27), as they shared the same manufacturer, but they were included as separate tests in our evaluation because of differences in the IFU leaflets. The cost of the tests varied between £1.89 (€2.19; $2.57) (T22) and £39.99 (T7).

**Table 1:** Test characteristics: Biomarkers, sample types, and indications for use

Test ID label	Product name (original cost)	Sample type		Biomarker	IFU clinical claim	Indication	Associated conditions	Symptoms for diagnosis	Characteristics to screen
T1	Menopause Test (£14.49)	Urine		FSH	Menopausal transition phase	Not stated	0	10	0
T2	Flourish Menopause Test Kit (£10.00)	Urine		FSH	Under menopausal process	Not stated	0	11	0
T3	Menopause (FSH) Rapid Test (£4.00)	Urine		FSH	Onset of menopause	Diagnosis	5	9	0
T4	FSH Rapid Menopause Test Midstream (£10.00)	Urine		FSH	Onset of menopause	Diagnosis	5	9	0
T5	SP-10 Male Fertility Rapid Test (£12.50)	Semen		Acrosomal protein SP-10	Low sperm concentration	Diagnosis, screening	0	1	0
T6	SpermCheck Fertility (£29.99)	Semen		Acrosomal protein SP-10	Low sperm concentration/count	Screening	0	0	0
T7	SwimCount Sperm Quality Test (£39.99)	Semen		Progressive motile sperm cells (cells/mL)	Predictor of male fertility	Not stated	0	0	0
T8	SURE CHECK HIV Self-Test (£33.95)	Capillary blood		Antibodies to HIV-1 and HIV-2	HIV infection	Screening	1	0	1
T9	Female Chlamydia STI Test Kit (£19.99)	Vaginal swab		Not stated	*Chlamydia trachomatis*	Not stated	9	0	0
T10	Women’s Intimate Self-test (£8.99)	Vaginal swab		Vaginal pH	Thrush, bacterial, or trichomoniasis infection	Diagnosis	0	7	0
T11	Canestest Self-test for Vaginal Infections (£7.50)	Vaginal swab		Vaginal pH	Bacterial vaginosis, thrush, trichomonas	Diagnosis	2	1	0
T12	Urine Infection Test (£14.49)	Urine		Protein, nitrite, and leucocytes		Diagnosis	0	6	0
T13	Bowel Health Test (£14.49)	Faeces		Human haemoglobin	Early stages of colon cancer	Not stated	5	0	2
T14	FOB Rapid Test (Faeces) (£10.00)	Faeces		Human haemoglobin		Not stated	6	0	0
T15	Prostate Health Test (£14.49)	Capillary blood		Prostate specific antigen	Indicator of prostate health	Not stated	4	0	2
T16	Stomach Ulcer Test (£14.49)	Capillary blood		*Helicobacter pylori* antibodies		Not stated	3	2	0
T17	Gluten Sensitivity Test (£17.49)	Capillary blood		Anti-tissue transglutaminase IgA antibodies		Not stated	2	9	0
T18	One step Strep A Swab test (£14.49)	Throat swab (tonsils)		Group A streptococcal antigen	Group A streptococcal infection	Diagnosis	6	2	0
T19	Flowflex Influenza A/B Rapid Test (Self-Testing) (£2.50)	Nasal swab		Influenza A antigen and influenza B antigen	Acute influenza type A and B viral infections	Diagnosis, screening	2	9	0
T20	Flowflex SARS-CoV-2 Antigen Rapid Test (Self-Testing) (£1.93)	Nasal swab		SARS-CoV-2 nucleocapsid antigen	Covid-19	Diagnosis, screening	0	8	1
T21	One step test for SARS-CoV-2 Antigen (Colloidal Gold) (£2.00)	Nasal swab		SARS-CoV-2 nucleocapsid antigen	SARS-CoV-2 infection	Diagnosis, screening	0	7	1
T22	STEPAHEAD COVID-19 Antigen Rapid Test Kit (Swab) For Self-Testing (£1.89)	Nasal swab		SARS-CoV-2 nucleocapsid antigen	SARS-CoV-2 infection	Not stated	0	1	1
T23	Microalbuminuria (MAU) Rapid Test Kit (Colloidal Gold) (£8.00)	Urine		Albuminuria	Diagnosis of chronic kidney injury (CKI)	Diagnosis	4	0	0
T24	TSH Rapid Test Cassette (£10.00)	Capillary blood		TSH	Hypothyroidism	Diagnosis, screening	0	8	0
T25	Ferritin Rapid Test Cassette (£8.00)	Capillary blood		Ferritin	Iron deficiency anaemia	Not stated	8	12	0
T26	Iron Deficiency (£8.00)	Capillary blood		Ferritin	Iron deficiency anaemia	Not stated	4	5	0
T27	Vitamin D Rapid Test Cassette (£8.00)	Capillary blood		25-hydroxyvitamin D	Vitamin D deficiency	Diagnosis, screening, monitoring	18	0	0
T28	Vitamin D Test (£8.00)	Capillary blood		25-hydroxyvitamin D	Vitamin D deficiency	Diagnosis, screening, monitoring	18	0	0
T29	Cholesterol Level Test (£14.99)	Capillary blood		Total cholesterol	Hypercholesterolemia	Not stated	7	0	0
T30	Blood Glucose Test (£12.99)	Capillary blood		Blood glucose	Blood sugar level	Not stated	8	0	0

£1.00 (€1.16; $1.36).

FOB=faecal occult blood; FSH=follicle stimulating hormone; IFU=instructions for use; IgA=immunoglobulin A; STI=sexually transmitted infection; TSH=thyroid stimulating hormone.

The 30 included tests covered 19 different clinical conditions and 20 different biomarkers: follicle stimulating hormone (T1-T4); acrosomal protein SP-10 (T5 and T6); progressive motile sperm cells (T7); antibodies to HIV-1 and HIV-2 (T8); vaginal pH (T10 and T11); urinary protein, nitrite, and leucocytes (T12); human haemoglobin (T13 and T14); prostate specific antigen (T15); *Helicobacter pylori* antibodies (T16); anti-tissue transglutaminase IgA antibodies (T17); group A streptococcal antigen (T18); influenza A antigen and influenza B antigen (T19); SARS-CoV-2 nucleocapsid antigen (T20-T22); urinary albuminuria (T23); thyroid stimulating hormone (T24); ferritin (T25 and T26); 25-hydroxyvitamin D (T27 and T28); total cholesterol level (T29); and blood glucose level (T30). One test, for chlamydia in women (T9), did not state the biomarker.

The 30 tests utilised seven different sample types, including faecal samples (two tests), finger-prick capillary blood (11), urine (six), semen (three), vaginal swabs (three), nasal swabs (four), and a throat swab (one). Seventeen used solid cassettes, four used test strips in cassettes, five used dipsticks, one used a blood spot, and three used bespoke devices.

### Information available on self-test boxes at point of sale

Information available at the point of sale, which is likely to guide consumers’ decisions to purchase a test, was assessed (table 2). All tests included a CE (Conformité Européene) mark on the box, and all but one (T2) included a statement that the test was designed for self-testing.

**Table 2:** Information provided at point of sale on self-test boxes

Test ID label	Health condition to be detected	Information about indications for testing	Biomarker stated	Advice after test	Claim about performance	Time to test result
T1	Menopause			Consult doctor	Reliable	3 minutes
T2	Menopause				Reliable	10 minutes
T3	Menopause		FSH		99% accurate	3 minutes
T4	Menopause		FSH		99% accurate	3 minutes
T5	Male fertility	Men	Sperm count		98% accurate	5 minutes
T6	Male fertility	Men. Trying to conceive	Sperm count	Test every 2/3 months	Proven accurate	Minutes*
T7	Sperm quality		Progressive motile sperm cells (PMSC)	Consult doctor or make lifestyle changes		30 minutes
T8	HIV viral infection	Not for those on antiretroviral treatment or in HIV vaccine trial	HIV infection	Confirming results with doctor		15 minutes
T9	Chlamydia in women	Women. Do not test during menstruation, pregnancy, or recent course antibiotics	Chlamydia infection			Minutes*
T10	Common vaginal infections	Women. One or more of itching, burning, pain when urinating, discharge, odour	Vaginal pH	Consult doctor or pharmacist	90% accurate	10 seconds
T11	Common vaginal infections	One or more of itching, burning, pain when urinating, discharge, odour	Vaginal pH	Seek attention for treatment	90% accurate	Seconds*
T12	Urine infections	One or more of burning when urinating, frequency, cloudy urine, odour, pain, fever	Protein, nitrite, leucocytes			
T13	Symptoms of colon polyps (polyps precede bowel cancer)					
T14	Bowel health (aid in the diagnosis of bowel cancer)		Occult blood		90% accurate	5 minutes
T15	Prostate health (enlargement of the prostate, prostatitis, urinary infection, early indication of prostate cancer)		PSA	Consult GP		10 minutes
T16	Stomach ulcer test		*H pylori*			10 minutes
T17	Gluten sensitivity test	Symptoms	TTG		97% accurate	15 minutes
T18	Streptococcus A detection		Strep A			
T19	Influenza A/B		Influenza A/B			
T20	SARS-CoV-2		SARS-CoV-2			
T21	SARS-CoV-2		SARS-CoV-2			
T22	Covid-19		Covid-19			
T23	None		Albumin		98% accurate	3 minutes
T24	Underactive thyroid		TSH		98% accurate	3 minutes
T25	Iron deficiency		Ferritin		95% accurate	5 minutes
T26	Iron deficiency		Ferritin			5 minutes
T27	Vitamin D deficiency		Vit D		Clinically tested accuracy	10 minutes
T28	Vitamin D deficiency		25-hydroxy Vit D			10 minutes
T29	Cholesterol level		Cholesterol level			3 minutes
T30	Glucose		Glucose			Minutes*

FSH=follicle stimulating hormone; PSA=prostate specific antigen; Strep=streptococcus; TSH=thyroid stimulating hormone; TTG=tissue transglutaminase.

* No specific duration specified.

Most of the test boxes included the sample to be used (26/30, 87%), information about the biomarker (27/30, 90%), and the health condition (29/30, 97%) to be detected. Information about the biomarker was judged to be written in inaccessible language for many of the tests, as it was based on technical terms (eg, occult blood, ferritin, *H pylori*, albumin, nitrite, leucocytes).

Indications about who should use a test are essential, as achieving the claimed test performance partly depends on whether users reflect the population in which the test was evaluated. Only eight of the 30 tests provided information about who should or should not use the test: four specified biological sex (T5, T6, T9, and T10), four specified the presence of symptoms (T10-T12 and T17), two provided information on who should not use the test (T8 and T9), and one specified if “trying to conceive” (T6).

Most of the test boxes (23/30, 77%) included information on the time to a test result, ranging from 10 seconds (T10) to 30 minutes (T7), emphasising that the tests provide results at the point of use. Less than half of the test boxes (14/30, 48%) included any statement about test accuracy. In four, information on test accuracy was in the form of statements such as reliable (T1 and T2), proven accurate (T6), and clinically tested accuracy (T27). In the remaining 10 tests, quantitative information about the test’s overall accuracy was presented. Information about action to be taken after the test was absent from most of the test boxes (23/30). Without this information, users cannot make informed decisions about whether and how purchasing the test might affect their care. For example, whether using the test might expedite treatment or reduce the need for consultation with a healthcare professional.

### Instructions for use leaflets

#### Clinical condition to be detected

Of the 30 tests, 29 made some statement about the clinical condition the test was designed to detect (table 1 and supplementary table A2), either implied by the name of the test (eg, urine infection test (T12), stomach ulcer test (T16), gluten sensitivity test (T17)) or in the IFU clinical claim. The clinical condition was sometimes vaguely and inconsistently described—for example, prostate specific antigen as an “indicator of prostate health” and as an “early indication of prostate cancer” (T15). Descriptions of clinical conditions to be detected were also inconsistent across tests for the same condition. For example, menopause tests variably stated that they could detect the menopausal transition phase (T1), menopausal process (T2), or onset of menopause (T3 and T4). The faecal occult blood rapid test (T14) states it is for the “detection of human occult blood in stool to aid in the diagnosis of bowel cancer,” whereas the bowel health test (T13) states that it is “to detect symptoms of colon polyps” and that “95% of all cases of colon (bowel) cancer result from benign tumours called polyps.” Nine tests (T5, T6, and T24-T30) state only that the test detects an abnormal level of the biomarker (deficiency, high values, or low values).

#### Test application

Sixteen tests explicitly stated whether the test was to be used for screening, diagnosis, or monitoring (table 1 and supplementary table A2), seven for diagnosis alone, two for screening alone, and seven for both diagnosis and screening, two of which also claimed to be used for monitoring. When we supplemented the explicit statements with our assessment of information supplied in the IFU leaflets (see supplementary table A2), we judged that 14 tests were recommended for diagnosis alone, eight for screening alone, five for both diagnosis and screening, and two for screening, diagnosis, and monitoring. No relevant information was available for one test (T9). Information that might guide test use, such as the presence of symptoms (for diagnosis) or risk factors (for screening), were not consistently placed in the IFU. The urine infection test (T12) provided contradictory messages, stating that it was for screening but listing symptoms that would indicate the need for use of the test.

#### Indications for use

For tests where diagnosis was either explicitly stated or implicitly implied as the intended application of a test, based on described symptoms, the descriptions varied from extensive, often non-specific symptoms, to vague or no information (see supplementary table A2). One covid-19 test stated the test was to be used if an individual had known or suspected covid-19 (T22), and one vaginal infection test stated it was to be used with abnormal vaginal symptoms (T11). Two vitamin D tests with an explicit diagnosis application provided no examples of symptoms that would prompt use (T27 and T28). Tests with the most extensive lists of symptoms were for menopause (9-11 symptoms) (T1-T4), iron deficiency (8-12 symptoms) (T26 and T27), and influenza (nine symptoms, with the list concluding with “and so on”) (T19).

Ten of 15 tests that explicitly or implicitly stated they were to be used for screening provided no information about indications (T6, T7, T14, T19, T23, T24, and T27-T30), whereas two merely stated no symptoms (T20 and T21). Three tests did not provide any indications for use but listed potential health conditions (ranging from rare to common and from benign to serious) that may be present in the event the test result indicated abnormality—for example, “rickets (children)” to “higher mortality, etc” for a vitamin D test (T27).

#### Interpretation of test results

For 20/30 (67%) tests (T1-T4, T8, T9, T12-T24, and T29), a higher value or the presence of a biomarker was associated with the presence of the health condition being tested. One male fertility test (T7) categorised results into two “normal” ranges but still functioned as a dichotomy regarding the target condition (normal sperm count levels), classifying sperm count as either normal or low. For five tests (T5-T7, T25, and T26), the health condition being tested for was associated with lower biomarker levels, the absence of a test (T) value on the lateral flow test indicating abnormal status. This is unintuitive and may be the opposite of what users expect, therefore the risk for misinterpretation should be mentioned in the instructions.

Interpreting the results in five of the 30 tests was more complex. For the two vaginal pH tests (T10 and T11) a combination of symptoms and the test result were required to distinguish between three different infections. In three tests (T27, T28, and T30), the results involved more than two categories. For vitamin D, results were categorised as deficient, insufficient, sufficient, or excess, whereas for blood glucose the results were categorised as below, within normal range, or above normal range.

Key to interpreting the clinical importance of a test result, particularly if the results are to inform further action in consultation with healthcare professionals, is knowing the threshold value above which the condition being tested for is indicated. Eighteen of the 30 tests provided thresholds in the IFU (T1-T7, T10, T14, T15, and T23-T30), five reported the limit of detection values (T18-T22), and seven provided no statement on the limit of detection or the threshold used to define an abnormal test result (T8, T9, T11-T13, T16, and T17) (see supplementary table A2).

### Recommended actions

In the IFU we assessed recommended actions as stated for healthcare consultations, further testing, and treatment or behaviour changes (table 3 and supplementary table A3). Twenty seven of 30 (90%) IFU leaflets recommended that positive (or abnormal) test results should be followed up with a healthcare professional (pharmacist (T10 and T11), doctor (T2-T4, T6-T9, T12-T18, T20-T22, and T24-T30), or medical expert (T5)). Three of the 30 (10%) tests provided no recommendations on further action, regardless of whether the test result was positive or negative (T1, T19, and T23). Fourteen of the 27 (52%) IFU leaflets also recommended seeking medical care if the test result was negative but symptoms (including inability to conceive) persisted (T6, T7, T10-T12, T16-T18, T22, and T24-T28), or for the HIV test (T8) in cases of recent exposure or within the 12 week seroconversion window. Six (20%) tests (T19 and T24-T28) stated positive and negative results should be assessed by a “secondary analytical method” (not otherwise specified) to confirm a result.

**Table 3:** Test interpretation and recommended actions

Test ID label: product name; result	Interpretation	Recommended medical contact	Treatment/actions	Further tests
**T1: Menopause Test**				
Positive	Assume that you have reached post menopause if both test results are positive			Repeat the test a week later if first is positive
Negative	You have not yet reached post-menopause			
**T2: Flourish Menopause Test Kit**				
Positive	You could be under menopausal process if both test results are positive	Gynaecologist		Repeat the test 5-7 days later if first is positive
Negative	You are probably not under menopausal process	Gynaecologist		Repeat test 40-60 days later if you still have symptoms
**T3: Menopause (FSH) Rapid Test**				
Perimenopausal symptoms:				
Positive	Most likely (two positive test results) or may be in perimenopause (one positive test)	Doctor	Do not immediately discontinue contraception	
Negative	Most likely not experiencing perimenopause	Doctor		Test can be repeated
Menopausal symptoms:				
Positive	Menopause has most likely occurred (after one positive test)			
Negative				Repeat testing in following month if still have symptoms
**T4: FSH Rapid Menopause Test Midstream**				
Perimenopausal symptoms:				
Positive	Most likely (two positive test results) or may be in perimenopause (one positive test)	Doctor	Do not immediately discontinue contraception	
Negative	Most likely not experiencing perimenopause	Doctor		Test can be repeated
Menopausal symptoms:				
Positive	Menopause has most likely occurred (one positive test)			
Negative				Repeat testing in following month if still have symptoms
**T5: SP-10 Male Fertility Rapid Test**				
Positive	Sperm concentration is less than 15 million/mL	Expert medical advice		
Negative	Sperm concentration is above 15 million/mL			
**T6: SpermCheck Fertility**				
Positive	Sperm count concentration is less than 15 million/mL	Doctor	Possible treatment for subfertility as advised by doctor	Complete semen analysis
Negative	Sperm count is at least 15 million/mL	Doctor if still experiencing fertility issues		Complete semen analysis and fertility evaluation for partner
**T7: SwimCount Sperm Quality Test**				
Positive	PMSC concentration is lower than for fertile men; may be unable to get your partner pregnant naturally	Doctor	Lifestyle changes to improve sperm concentration	Repeat test in three months after lifestyle changes
Negative	PMSC concentration is normal; high chance making partner pregnant naturally	Doctor if still experiencing fertility issues		
**T8: SURE CHECK HIV Self-Test**				
Positive	Probably HIV positive	Doctor	Protect yourself and others	Confirmatory laboratory test
Negative	Probably HIV negative	Doctor if possibility of being in the seroconversion period		Repeat the test in 3 months if possible exposure to HIV
**T9: Female Chlamydia STI Test Kit**				
Positive		Doctor	Sexual partner should also take a test and undergo treatment	
Negative	Highly unlikely to have chlamydia			Regular testing recommended
**T10: Women’s Intimate Self-test**				
Positive	Vaginal pH is higher than normal; probably have bacterial vaginosis/trichomoniasis dependent on symptoms	Pharmacist if bacterial vaginosis suspected; doctor if trichomoniasis suspected	Product for bacterial vaginosis from pharmacist; treatment for trichomoniasis from doctor	
Negative	Vaginal pH is normal; probably have thrush if symptoms are present	Pharmacist if thrush is suspected	Products for thrush advised by pharmacist	
**T11: Canestest Self-test for Vaginal Infections**				
Positive	Vaginal pH is higher than normal; probably have bacterial vaginosis/trichomoniasis infection dependent on symptoms	Pharmacist if bacterial vaginosis suspected; doctor if trichomoniasis suspected or if no symptoms	Treatment for bacterial vaginosis from pharmacist or doctor; trichomoniasis from doctor	
Negative	Vaginal pH is normal; probably have thrush if symptoms are present	Pharmacist if thrush suspected	Over-the-counter thrush products	
**T12: Urine Infection Test**				
Positive	Likely that something is wrong with your urine	GP		Further investigation by GP
Negative		Doctor if symptoms persist		
**T13: Bowel Health Test**				
Positive	Blood detected in stool	Doctor		
Negative	No blood detected in stool (does not exclude possibility of bowel condition)			Test yourself or arrange to be tested annually from age 40 years
**T14: FOB Rapid Test (Faeces)**				
Positive	Blood in faeces does not necessarily indicate colorectal bleeding	Doctor		
Negative				
**T15: Prostate Health Test**				
Positive	PSA level is higher than normal (4 ng/mL)	Doctor		
Negative	PSA level is in the normal range			Regular testing if between 50 and 75 years of family history
**T16: Stomach Ulcer Test**				
Positive	*H. pylori* antibodies detected	Doctor		
Negative	No *H. pylori* antibodies detected	Doctor if symptoms persist		
**T17: Gluten Sensitivity Test**				
Positive	Anti-t-TG IgA type antibodies are present	Doctor		
Negative	No anti-t-TG IgA type antibodies detected	Doctor if symptoms persist		
**T18: One step Strep A Swab test**				
Positive	May be in a stage of Strep A infection	Doctor		
Negative	Concentration of Strep A antigen is zero or below detection limit (probably not infected)	Doctor if symptoms persist or experience serious complications		
**T19: Flowflex Influenza A/B Rapid Test (Self-Testing)**				
Positive	Influenza A or B antigen detected			Alternative confirmatory tests
Negative	No influenza or A/B antigen detected (does not preclude influenza or other infections)			Alternative confirmatory tests
**T20: Flowflex SARS-CoV-2 Antigen Rapid Test (Self-Testing)**				
Positive	Presence of SARS-CoV-2 antigen; very likely infected with covid-19	Doctor	Follow self-isolation rules; contact local health department	PCR test
Negative	Unlikely to currently have covid-19; infection may still be present (does not rule out other infections)		Follow current rules	Repeat test after 1-2 days if infection suspected; molecular assay if necessary
**T21: One step test for SARS-CoV-2 Antigen (Colloidal Gold)**				
Positive	Presence of SARS-CoV-2 antigen; likely to have covid-19	Doctor	Follow self-isolation rules; contact local health department	Molecular testing
Negative	No SARS-CoV-2 antigen detected; unlikely to be infected with covid-19; there may still be an infection		Follow current rules	Repeat test after 1-2 days if infection suspected or with a molecular assay
**T22: STEPAHEAD COVID-19 Antigen Rapid Test Kit (Swab) For Self-Testing**				
Positive	Presence of SARS-CoV-2; suspicion of covid-19 infection	Doctor	Follow self-isolation rules; contact local health department	PCR test
Negative	Does not preclude SARS-CoV-2 infection; there may still be an infection	Healthcare provider if still experiencing covid-like symptoms	Follow current rules	Repeat test after 1-2 days if infection suspected
**T23: Microalbuminuria (MAU) Rapid Test Kit (Colloidal Gold)**				
Positive	Albumin in urine is more than 20 mg/L (does not necessarily indicate kidney injury)			
Negative	Albumin in urine is less than the cutoff value			
**T24: TSH Rapid Test Cassette**				
Positive	TSH level is higher than normal (5 μIU/mL) (indicates an underactive thyroid (hypothyroidism))	Physician; repeatedly abnormal results discussed with doctor		Further tests decided by physician; quantitative laboratory TSH assay
Negative	TSH below 5 μIU/mL within normal range (not in range to consider hypothyroidism; hyperthyroidism cannot be excluded)	Physician if symptoms persist		Quantitative laboratory TSH assay
**T25: Ferritin Rapid Test Cassette**				
Positive	Ferritin concentration in blood is lower than 30 ng/mL; possible iron deficiency	Physician; repeatedly abnormal results discussed with doctor		Tests decided by physician; use a secondary analytical method
Negative	Ferritin higher than 30 ng/mL and within normal range; no potential iron deficiency	Physician if symptoms persist		Use a secondary analytical method
**T26: Iron Deficiency**				
Positive	Ferritin concentration in blood is lower than 30 ng/mL; possible iron deficiency	Physician; repeatedly abnormal results discussed with doctor		Tests decided by physician; use a secondary analytical method
Negative	Ferritin higher than 30 ng/mL and within normal range; no potential iron deficiency	Physician if symptoms persist		Use a secondary analytical method
**T27: Vitamin D Rapid Test Cassette**				
Positive	Vitamin D level is less than 30 ng/mL (deficient or insufficient)	Physician if result is deficient, insufficient, or excess	Vitamin D supplements	Tests decided by physician; use a secondary analytical method
Negative	Vitamin D level is higher than or equal to 30 ng/mL and within normal range	Physician if symptoms persist		Use a secondary analytical method
**T28: Vitamin D Test**				
Positive	Vitamin D level is less than 30 ng/mL (deficient or insufficient)	Physician if result is deficient, insufficient, or excess	Vitamin D supplements	Tests decided by physician; use a secondary analytical method
Negative	Vitamin D level is higher than or equal to 30 ng/mL and within normal range	Physician if symptoms persist		Secondary analytical method
**T29: Cholesterol Level Test**				
Positive	Cholesterol level is probably high if above 5.2 mmol/L	Doctor	Treatment as advised by doctor	Further tests as decided by doctor
Negative	Cholesterol is probably normal if level is less than 5.2 mmol/L			
**T30: Blood Glucose Test**				
Positive	Below 3.9 or above 6.1 mmol/L blood sugar level is outside the normal range	Doctor if both tests are abnormal		Repeat test on a different day; detailed assessment by doctor

Anti-t-TG=anti-tissue transglutaminase FOB=faecal occult blood; PMSC=progressive motile sperm cells; PCR=polymerase chain reaction; PSA=prostate specific antigen; STI=sexually transmitted infection; TSH=thyroid stimulating hormone.

Twelve (40%) tests (seven test types) recommended repeat testing after a negative test result. The three covid-19 tests (T20-T22) recommended repeat testing 1-2 days after a negative test result, whereas one also suggested “re-testing with a (more sensitive) molecular test (PCR) [polymerase chain reaction]” (T21). The HIV test (T8) recommended retesting 12 weeks after an initial negative test result owing to the potential for delayed seroconversion. Three of the four menopause tests (T2-T4) recommended retesting between one month and 60 days after a negative test result in the continued presence of symptoms. Both vitamin D tests (T27 and T28) indicated that the test could be used to monitor vitamin D levels to decide whether vitamin D supplements should be taken. The chlamydia test (T9) recommended “regular” testing, with no further details. One of two faecal occult blood tests (T13) recommended “annual use of the test over the age of 40,” and the prostate cancer test (T15) recommended testing “regularly” between ages 50 and 75 years or in the case of a “family history.”

Seven tests indicated treatment or health behaviour changes. The two vaginal infection tests (T10 and T11) recommended treatment should be obtained after a positive test result either over the counter or from a doctor, depending on the type of infection detected. The chlamydia test (T9) suggested that sexual partners should be tested if the test result was positive, and the HIV test (T8) suggested protection should be used if the test result was positive. The three covid-19 tests (T20-T22) all stated that rules and protective measures should be followed if the test results were positive. Two of the four menopause tests explicitly stated that results should not be used as the basis for discontinuing contraception (T3 and T4).

### Coherence of marketing for self-tests with UK guidance

With the exception of SARS-CoV-2, UK guidance does not endorse self-testing for the 19 clinical conditions covered in our sample.^12^ ^13^ ^14^ ^15^ ^16^ ^17^ ^18^ ^19^ ^20^ ^21^ However, for seven (37%) of the 19 conditions (HIV, vaginal infections, prostate health, SARS-CoV-2, gluten sensitivity, influenza, and Streptococcus A) covered by 10 tests (T8, T10, T11, T15, and T17-T22) we judged that the marketed indication for testing was coherent with relevant UK guidance. For example, UK guidance states that men older than 50 years can request a prostate specific antigen test from their general practitioner,^16^ and the prostate health test (T15) states that “prostate cancer is less common in men below the age of 50.”

One of the three male fertility tests (T5) recommended use after one year of trying to conceive, which is coherent with UK guidance,^22^ whereas the other two did not provide guidance on when the test should be used (T6 and T7).

For 11 of the 19 (58%) conditions (covered by 14 tests), the intended use as marketed was judged to be contrary to UK or WHO guidelines because of one or more of: use in more restricted populations in terms of sex, age, or indication (menopause (T1-T4),^23^ chlamydia in women (T9),^24^ ^25^ *H pylori* (T16),^26^ vitamin D (T28),^27^ cholesterol (T29),^28^ ^29^ blood glucose (T30),^30^ and urine infection (T12)^31^); use at a specified frequency (faecal occult blood (T13 and T14)^32^); use at a different threshold (faecal occult blood (T13 and T14)^32^); and the decision to test being complex and/or the need to be integrated into a testing strategy (urine infection (T12),^31^ iron deficiency (T26),^33^ microalbuminuria (T23),^34^ thyroid stimulating hormone (T24)^35^ ^36^). For example, NICE guidelines state that tests for follicle stimulating hormone should not be used in women older than 45 years (T1-T4)^23^ and it does not recommend routine testing for vitamin D deficiency in asymptomatic individuals (T27 and T28).^27^ The UK National Screening Committee recommends use of a faecal immunochemical test every two years in men and women aged 50 to 74 years at a threshold of 120 μg/g in England and 60 μg/g in Scotland and Wales^32^; compared with the 50 μg/g for the faecal occult blood self-tests (T13 and T14). NICE guidelines for managing dyspepsia^26^ are to assess and exclude other causes before considering drugs and testing for *H pylori* (T16). NICE^33^ recommends a full blood count before testing for ferritin, and the decision to test thyroid stimulating hormone levels (T24) should take into account the nature and severity of symptoms, population to be tested (adults, children, pregnant people), and the presence of co-existing conditions; thyroid stimulating hormone levels can be abnormal because of co-existing conditions.^35^ ^36^

## Discussion

A wide range of direct-to-consumer self-tests is available, indicating consumer demand, and they are not limited to specific healthcare problems. Thirty self-tests for the detection of 19 different conditions were available within a 10 mile radius of the University of Birmingham’s campus at Edgbaston. Tests were widely available for purchase across supermarkets and pharmacies. It was beyond the scope of this study to fully assess the market forces behind this widespread availability at the present time; however, enhanced curiosity about health, the immediacy of results, privacy concerns, and difficulty accessing healthcare are all factors that may influence consumers’ decisions to purchase and use self-tests. The tests required samples from nasal and oral pharyngeal secretions, capillary blood, urine, seminal fluid, vaginal secretions, and stool, showing the variety of body fluids acceptable for use.

Information provided by the tests in our sample is unlikely to be sufficient to support appropriate use. Of the 30 tests, only eight provided information on the box about who should or should not use the test, only seven indicated the action that should follow the result, and only 10 provided quantitative information about the accuracy of the test.

The purpose of the 30 tests was poorly stated in many of the IFU leaflets. Only 16 explicitly stated that the test was for screening, diagnosis, or monitoring (six for diagnosis only, three for screening only, seven for both diagnosis and screening, and two that could also be used for monitoring). Nine of the 30 tests did not indicate the symptoms or risk factors for their use, and the intended use was poorly justified, while some grouped symptoms with conditions associated with the condition being tested for, listing up to 18 vaguely related conditions.

The potential for direct-to-consumer self-tests to support self-care is unlikely to be realised by the tests currently marketed. Only two tests led to a recommendation for treatment, and five to a health behaviour change. However, nearly all the tests recommended follow-up with a healthcare professional (27/30 (90%) if test results were positive or abnormal, but also 14/30 (47%) if test results were negative). Indeed, most (21/30, 70%) of the tests did not progress or close care pathways: nine either recommended further confirmatory testing regardless of the test result or did not provide recommendations for action after positive or negative test results, and 12 recommended repeat testing at variable intervals after a negative test result.

The current marketing of self-tests is not coherent with evidence based guidance and there seems to be no attempt to facilitate integration of these tests with healthcare services. The use of the biomarkers for 11 of the 19 (58%) conditions considered were judged contrary to UK or WHO guidance because of the population, frequency, or test threshold, or because investigation of the clinical condition should be based on integrated testing strategies and not a single test. For at least one test where the threshold was reported, this differed from the threshold used in UK national screening programmes, although this was not explicitly reported in the IFU. Achieving coherence in recommendations about frequency and thresholds with national and international guidelines is unlikely because of the cost effectiveness for healthcare systems. However, lack of coherence about the role of a test in a clinical testing strategy is unlikely to be explained by cost considerations. Thresholds were not reported for 12 of the 30 (40%) tests, and no test provided information about the reliability of positive test results separate from negative test results. Absence of information about thresholds and performance in self-tests prevents healthcare professionals from drawing meaningful conclusions from public use and is likely to lead to further testing by healthcare services.

### Strengths and weaknesses of this study

Despite current concerns about self-tests expressed by multiple stakeholders representing clinical, pharmaceutical, laboratory, and policy sectors,^37^ ^38^ we are unaware of other cross sectional studies of self-tests available in high street shops in the UK. The tests in our sample covered a wide range of clinical conditions, ranging from relatively minor conditions (eg, vitamin D deficiency) to more serious conditions (eg, HIV), and the price range was wide (£1.89 to £39.99). We believe the tests we studied are indicative of the range of self-tests available in high street shops in the UK, as our sample included tests sold through most of the UK supermarkets, corporate pharmacies, and health and wellbeing shops. Although we were unable to include tests sold only in independent pharmacies, which have 25% of the market share, it is unlikely that those tests will differ from those sold in corporate pharmacies. We excluded tests used for pregnancy and ovulation, and for drug testing, including alcohol breath tests. This was in part a pragmatic decision, but we recognise that the quality of information for these tests is of interest to consumers and would also benefit from scrutiny.

Data extraction was checked by a second, independent person, using verbatim quotes from packages and IFU leaflets, and by a multidisciplinary team including representation from experts in regulation, clinical and public health practice, clinical epidemiology, and statistics, and test manufacturers. Although this facilitated breadth of interpretation, it could have resulted in overestimation of the accessibility of information available to lay people to support decision making after test use. Our sample of tests was obtained two years ago. Although it is possible that the quality of the information sold with self-tests changed during that period, we consider this unlikely in the absence of new regulation or guidance. As described in our paired paper,^10^ a recent (December 2024) check of self-tests for sale in the same area revealed an approximate doubling in the number of self-tests for sale but with little change in the range of health conditions represented. Most of the additional tests were found to be clones of tests already identified, produced by the same manufacturer, but sold under various names by different distributors.

### Explanations and implications for the public, clinicians, and policy makers

For a test to provide helpful information, it should be used at an appropriate point in a clinical pathway when a test result can influence decision making. Even a test with acceptable performance in a specific clinical context can result in inappropriate clinical decisions, and, at worst, may jeopardise safety if used correctly but in the wrong person for the wrong reason.

In the UK, misleading or non-evidence based promotion of medical devices can result in regulatory enforcement.^39^ However, the advertising of in vitro diagnostics is currently not regulated through specific UK legislation and is only subject to general laws about consumer advertising. This contrasts with the marketing of pharmaceuticals, which is overseen by the Proprietary Association of Great Britain, an industry self-regulatory body (https://www.pagb.co.uk/about-pagb/).^40^

Recent studies have shown how presentation of evidence can change consumers’ interest in using self-tests,^41^ and difficulties encountered by the public and professional understanding of medical test evidence is well rehearsed.^42^ ^43^ ^44^ Individuals within our patient and public involvement group considered CE marking an indicator of the trustworthiness of self-tests, and our findings suggest that confidence in CE marking as an indicator of quality and safety is misplaced. Further research is needed on what information is required by the public and in what format to support informed decision making.

A recent report from the Royal Statistical Society^45^ highlighted major concerns about the way in vitro diagnostics are currently evaluated and regulated. In vitro diagnostics themselves are currently regulated under the Medical Devices Regulations^39^ in Great Britain and the In Vitro Medical Devices Regulation (IVDR)^46^ ^47^ in the EU (transitioning from the previous In Vitro Medical Devices Directive^48^ ^49^), according to the risk of harm and the severity of that harm both to individuals and to the public. The risks associated with self-testing are distinguished in legislation, but compliance and consistent and correct interpretation are likely to be undermined by imprecise terminology and lack of detailed guidance. For example, regarding protection against risks associated with self-testing devices, the IVDR^46^ and relevant harmonised EU standards for labelling^50^ developed for manufacturers to show compliance with IVDR, state that “information and instructions provided by the manufacturer shall be easy for the intended user to understand and apply” and “the procedure [for performing the IVD examination] shall be written using simple terms that can be clearly understood by a lay person,” respectively. In addition to risks associated with self-testing, risk classification of tests for the purposes of conformity assessment^46^ ^47^ ^51^ are determined based on the manufacturer’s stated intended purpose of the test, which, as our study has shown, is often ill defined. Absent or vague indications for use observed in our sample encourage indiscriminate testing, which is associated with a higher risk of false positive results and subsequent unnecessary testing and treatment.^52^ Paradoxically, self-testing as an initiative to promote self-care is likely to place further demands on an already stretched healthcare system.^2^ ^3^ ^4^ The absence of clear indications for the use of self-tests may also undermine the practice of evidence based testing and may discourage individuals from engaging with population based screening programmes. Furthermore, a lack of clarity about the clinical condition being tested for may obscure the clinical importance of test results and the potential harms from test errors—for example, the test advertised as the “bowel health” test to detect “early stages of colon cancer” (T13) and the faecal occult blood rapid test to detect “occult blood” (T14).

Similarly, regarding decisions after self-testing, the IVDR lacks clarity, requiring that information for use must include “a statement clearly directing that the user should not take any decision of medical relevance defined, without first consulting the appropriate healthcare professional.”^46^ A lack of definition of “medical relevance” leads to the potential for misuse and undermines the ability of self-tests to support autonomy and self-care.

A demand for self-tests appears to exist, which suggests a potential unmet health need. Only one third of our patient and public involvement participants in 2023 were aware that self-tests were available on the market, but two thirds said they would consider buying one themselves. In this context, the model in which all testing is directed by a healthcare professional needs to be evaluated. Self-tests are likely to play an increasing role in healthcare both nationally^53^ and globally,^54^ with individuals encouraged to take greater control of their health to reduce healthcare consultations and effect positive change on individual health and health services.^8^ ^9^ A mixed market of over-the-counter and pharmacy regulated self-tests, as exists for pharmaceuticals, is a possible regulatory change that would enable the potential benefits of self-tests to be realised while mitigating inappropriate and harmful use. Consideration of self-tests within current NHS pathways, or indeed newly devised NHS pathways, could enable high quality tests to be endorsed and promoted. Self-tests have the potential to reduce inequalities by providing access to testing that is indicated for those unable or choosing not to attend traditional healthcare services. However, self-tests also have the potential to widen inequalities by providing testing based on the ability to pay rather than on clinical need, and the possibility for exploitation of vulnerable population groups.

### Conclusions

The current market and regulatory situation for direct-to-consumer self-tests are likely to make it difficult for the public to make informed decisions about when and how to use them, as well as how to interpret the findings and what subsequent actions should be taken. Clinicians working downstream of self-tests are likely to face major challenges in interpreting results, which may lead to increased costs for the patient or the healthcare system. If self-tests are to be embraced as a self-care initiative, supporting the public to decide when self-care is appropriate and when professional help is needed, evaluation and regulation requires development. Processes for the evaluation and regulation of self-tests are complex, and no single stakeholder group can robustly deal with the challenges described. Engagement with gatekeepers such as the Medicines and Healthcare Products Regulatory Agency, NICE, and the UK National Screening Committee is essential for the development of guidance around evaluation and use of self-tests. The British Standards Institute has a role as the UK national standards body in producing guidance for operationalisation of national and international legislation. Discussion with manufacturers, consumers, and healthcare professionals is equally important for sharing good practice, such as for existing guidance for pharmacies on the use of point-of-care testing,^55^ and for identifying key challenges and solutions. Given the complexity associated with ensuring the availability of safe and effective self-tests, an urgent need exists to establish guidance covering the evaluation (including usability, consumer and health service impact assessment), regulation, marketing and post-market surveillance of self-tests and to identify priorities for further research. Ensuring that patients are provided with tests that have been evaluated for clinical utility and patient usability is vital.

**What is already known on this topic**

Self-testing without the need for healthcare professional involvement is increasingly popular, with a wide range of tests available to consumers in the UK
Regulation specifically dealing with the marketing, evaluation, and use of self-tests is limited
Concerns have been raised by various stakeholders (clinicians, policy makers, statisticians) about the quality, appropriateness, and safety of self-tests, but no comprehensive study has examined what is currently available in high street shops in the UK

**What this study adds**

The current UK self-test market fails to support informed consumer use; most tests lack essential information about who should use them, how to interpret results, and what actions to take next
Many self-tests on the UK market contradict evidence based clinical guidance, particularly for intended population, testing frequency, test threshold, and post-test decision making, creating risks for misinterpretation and inappropriate healthcare decisions
The effectiveness of regulatory oversight is a serious concern, and this study highlights an urgent need for coherent guidance and improved regulation to protect both individuals and healthcare systems from misuse and misinformation

## Data Availability

No additional data available.

## Web extra

Extra material supplied by authors

Supplementary information: Appendix tables A1-A3

## Notes

Contributors: JJD, AR, and CD conceptualised the project. JJD obtained funding and supervised and managed the project. JJD, AR, BH, and CD designed the study and its methodology. AR, BH, CD, JJD, AUK, JA, KS, RA, SWB, and TM took part in the data curation, formal analysis, investigation, and writing of the original draft, and reviewing the final manuscript. CD and AR are joint first authors. JJD acts as guarantor. The corresponding author attests that all listed authors meet authorship criteria and that no others meeting the criteria have been omitted.

Transparency: The lead author affirms that the manuscript is an honest, accurate, and transparent account of the study being reported; that no important aspects of the study have been omitted; and that any discrepancies from the study as originally planned (and, if relevant, registered) have been explained.

Dissemination to participants and related patient and public communities: The results of this study have been shared with the Medicines and Healthcare Products Regulatory Agency, which informed the 2025 regulating medical devices in the UK regulation. The results have also been shared with the Royal Pharmaceutical Society, General Pharmaceutical Council, and Royal College of General Practitioners for dissemination to their members and also presented directly to clinicians and researchers at national diagnostics meetings. The work will be disseminated to the public through a public webinar and through traditional media and social media channels.

Provenance and peer review: Not commissioned; externally peer reviewed.
