# Supplementary material for: Direct-to-consumer self-tests sold in the UK in 2023: cross sectional review of information on intended use, instructions for use, and post-test decision making
Source: BMJ. 2025 Jul 23;390:e085546. doi: 10.1136/bmj-2025-085546 (PMC12284942; doi:10.1136/bmj-2025-085546)
Supplement: Supplementary file 1 — Supplementary information: Appendix tables A1-A3 [file davc085546.ww1.pdf]

Direct-to-consumer self-tests sold in the UK in 2023: an investigation into information about intended use, instructions for use and post-test decision-making

Appendix tables

Contents

Table A1: Questions asked in the public engagement research showcase event 1

Table A2: Details about intended use from Instructions for Use (IFU) sheets 2

Table A3: Details about test interpretation and recommendations following test results 6

Table A1: Questions asked in the public engagement research showcase event

Questionnaire for healthcare workers	Response options
1. Have you used lateral flow self-tests for COVID-19?	Yes / No
2. Have you used self-tests for any other conditions (except excluded tests, e.g. pregnancy, ovulation)?	Yes / No
3. Are you aware these self-tests are available for the general public to buy? (except COVID tests)	Yes / No
4. Would you ever consider buying one of these self-tests for yourself? (except COVID tests)	Yes / No / Not sure
5. Have any of your patients mentioned using self-tests (except for COVID)?	Yes / No
6. Would you trust the results of these self-tests?	Yes / No / Not sure
7. Do you think these self-tests have an overall positive or negative effect on the public?	Positive / Negative / Balanced
8. Do you think these self-tests have an overall positive or negative effect on health services?	Positive / Negative / Balanced
9. Do you have a positive, negative, or neutral view of these self-tests?	Positive / Negative / Neutral
10. Do you think the 'quality' of these self-tests are adequate for their use?	Yes / No / Not sure
11. Do you have any personal experience regarding these self-tests?	Free text
**Questionnaire for lay-people**	**Response options**
1. Have you used lateral flow self-tests for COVID-19?	Yes / No
2. Have you used self-tests for any other conditions (except excluded tests, e.g. pregnancy, ovulation)?	Yes / No
3. Are you aware these self-tests are available for the general public to buy? (except COVID tests)	Yes / No
4. Would you ever consider buying one of these self-tests for yourself? (except COVID tests)	Yes / No / Not sure
5. After looking at the boxes... Do you think the information on the boxes is sufficient? (Prompt - to help the consumer understand what the test does, who should buy the test and when they should take the test, etc.)	Yes / No / Not sure
6. Would you trust the results of these self-tests?	Yes / No / Not sure
7. Do you think these self-tests have an overall positive or negative effect on the public?	Positive / Negative / Balanced
8. Do you think these self-tests have an overall positive or negative effect on health services?	Positive / Negative / Balanced
9. Do you have a positive, negative, or neutral view of these self-tests?	Positive / Negative / Neutral
10. Do you think the 'quality' of these self-tests are adequate for their use?	Yes / No / Not sure
11. Do you have any personal experience regarding these self-tests?	Free text

Table A2: Details about intended use from Instructions for Use (IFU) sheets

Test ID label	Test Product Name	Claim made on box	Indication for Use statement	IFU technical claim *(Test threshold)*	IFU clinical claim	Outcomes associated with health condition being tested for	Implied Test Application (Screening, Diagnosis or Monitoring)	History or symptoms if for diagnosis	Population characteristics if for screening
T1	Menopause Test	A simple test to determine whether you have had your last period and reached the post menopause stage.<br><br>(identify whether you have gone through the menopausal transition)	Information<br><br>*No use statement*	detection of elevated FSH levels *(25mlU/mL and above)*	identify whether you have gone through the menopausal transition phase	*No detail*	Symptomatic diagnosis	hot flushes<br>vaginal itching<br>sweating<br>insomnia<br>depression<br>heart problems<br>reduced libido<br>dry eyes<br>painful joints<br>sluggish digestion	*No detail*
T2	Flourish Menopause Test Kit	Specially designed for testing the menopause	Summary and Explanation<br><br>*No use statement*	detect the presence of FSH in urine *(25mlU/mL and above)*	*None stated in Summary and Explanation*<br><br>determine whether you are under menopausal process (Result Interpretation)	*No detail*	Symptomatic diagnosis	irregular menstrual cycle duration<br>hot flushes<br>insomnia<br>palpitations<br>mouth ulcers<br>gingivitis<br>hair loss<br>dry vagina<br>osteoporosis<br>chills<br>excessive sweating	*No detail*
T3	Menopause (FSH) Rapid Test	Detection of follicle-stimulating hormone (FSH) in urine to aid in the detection of menopause.	Intended Use statement<br><br>Evaluate the onset of menopause	follicle stimulating hormone (FSH) *(25mlU/mL and above)*	evaluate the onset of menopause in women	osteoporosis<br>increased blood pressure<br>increased cholesterol<br>increased risk of heart disease	Symptomatic diagnosis	hot flushes<br>irregular menstrual cycles<br>sleep disorders<br>vaginal dryness<br>hair loss<br>anxiety<br>mood swings<br>short-term memory loss<br>fatigue	*No detail*
T4	FSH Rapid Menopause Test Midstream	Detect levels of FSH (follicle-stimulating hormone) as an indicator of onset of menopause	Intended Use statement<br><br>Aid in the detection of menopause	follicle stimulating hormone (FSH) *(25mlU/mL and above)*	evaluate the onset of menopause in women	osteoporosis<br>increased blood pressure<br>increased cholesterol<br>increased risk of heart disease	Symptomatic diagnosis	hot flushes<br>irregular menstrual cycles<br>sleep disorders<br>vaginal dryness<br>hair loss<br>anxiety<br>mood swings<br>short-term memory loss<br>fatigue	*No detail*
T5	SP-10 Male Fertility Rapid Test	To determine sperm count as an indicator of male fertility.	Intended Use statement<br><br>Assist diagnosis<br>Curative effect	acrosomal protein SP-10 sperm concentration *(15 million/mL and above)*	sperm concentration …effect male infertility and guidance for reproductive planning.	*No detail*	Symptomatic diagnosis	failure to become pregnant after one year of unprotected, well-timed intercourse	*No detail*
T6	SpermCheck Fertility	Indicates fertility sperm-count level.<br><br>(Results show whether a man's sperm count is within or below a healthy threshold for male fertility)	Indications For Use statement<br><br>Screening test	concentration of sperm in semen *(15 million/mL and above)*	sperm concentration/count as an indicator of Male fertility	*No detail*	Screening	*No detail*	*No detail*
T7	SwimCount™ Sperm Quality Test	Measures your sperm quality and their ability to swim.<br><br>(It measures the concentration of Progressive Motile Sperm Cells (PMSCs) in your semen. The number if PMSCs is the best predictor for male fertility and a key factor in achieving pregnancy).	*No statement*<br><br>*No use statement*	concentration of swimming (Progressive Motile) Sperm Cells *(PMSC/mL: low (<5 million), Normal-Mid (5-20 million), Normal-High (>20 million))*	best predictor of male fertility and a key factor in achieving pregnancy.	*No detail*	Screening	*No detail*	*No detail*
T8	SURE CHECK® HIV Self-Test	Screening test for HIV (the virus responsible for AIDS).	Read Me Now statement<br><br>Screening test	Antibodies to HIV-1 and HIV-2 *(no threshold stated)*	HIV infection	AIDS	Screening		Exposure to HIV
T9	Female Chlamydia STI Test Kit	Detect infection with Chlamydia trachomatis<br><br>(Inflammation of the fallopian tubes (salpingitis), inflammation of the womb (cervicitis), pelvic	General Information<br><br>*No use statement*	*No statement (no threshold stated)*	Chlamydia trachomatis	eye infections<br>arthritis<br>urinary tract infections<br>infertility<br>ectopic pregnancy<br>premature births	*Not stated*	*No detail*	*No detail*

Test ID label	Test Product Name	Claim made on box	Indication for Use statement	IFU technical claim *(Test threshold)*	IFU clinical claim	Outcomes associated with health condition being tested for	Implied Test Application (Screening, Diagnosis or Monitoring)	History or symptoms if for diagnosis	Population characteristics if for screening
		inflammatory disease (PID) and infertility).				blindness in newborns<br>conjunctivitis in newborns<br>pneumonia in newborns			
T10	Women's Intimate Self-test	Helps to identify common vaginal infections and find the appropriate treatment.<br><br>(possible thrush, possible bacterial or trichomoniasis)	Opening paragraph<br><br>Identify common infections	vaginal pH values<br>*(pH values over >4.4 are higher than normal)*	Thrush, bacterial or trichomoniasis infection	*No detail*	Symptomatic diagnosis	thick white cottage cheese discharge<br>fishy smell<br>white or grey liquid discharge<br>foamy yellow discharge<br>greenish discharge<br>itching/burning<br>pain when urinating	*No detail*
T11	Canestest Self-test for Vaginal Infections	Helps to diagnose common vaginal infections and to find the right treatment.<br><br>(cystitis, severe infections of the kidney)	Opening paragraph<br><br>Diagnosis	vaginal pH values<br>*(no threshold stated)*	Bacterial Vaginosis, Vaginal Thrush, Trichomonas	complications in pregnancy<br>complications in childbirth	Symptomatic diagnosis	abnormal vaginal symptoms	*No detail*
T12	Urine Infection Test	Detect indirect signs of infection such as protein, leucocytes, nitrites.	General Information<br><br>*No use statement*	protein, leucocytes, nitrites<br>*(no threshold stated)*	*No statement*	*No detail*	Symptomatic diagnosis	sensation of burning<br>strong urge to urinate<br>cloudy urine<br>urine with strong colour<br>pain in the lower back<br>fever and shivering	*No detail*
T13	Bowel Health Test	Detect symptoms of colon polyps<br><br>(Early detection of colon cancer is very important and everyone over the age of 40 should perform a yearly faecal occult blood test in order to minimise risk).	General Information<br><br>*No use statement*	Blood in stool that is not yet visible<br>*(no threshold stated)*	Early stages of colon cancer	colon polyps<br>intestinal diseases<br>inflammatory bowel conditions<br>haemorrhoids<br>changes in digestive tract	Screening	*No detail*	Aged over 40, or under 40 if there is a family history of colon cancer or polyps
T14	FOB Rapid Test (Faeces)	*No clear claim*<br><br>(Detection of human occult blood in stool, to aid in the diagnosis of bowel cancer)	Intended Use statement<br><br>*No use statement*	Occult blood<br>*(blood at 50 ng/ml or higher or 6 µg/g faeces)*	*No statement*	bowel cancer<br>ulcers<br>polyps<br>colitis<br>diverticulitis<br>fissures	Screening	*No detail*	*No detail*
T15	Prostate Health Test	Detect abnormal levels of Prostate Specific Antigen (PSA)<br><br>(can be due to conditions such as enlargement of the prostate gland, prostatitis, or urinary infection. It may also be a cause of prostate cancer).	General Information<br><br>*No use statement*	Prostate Specific Antigen (PSA)<br>*(4ng/ml or above)*	indicator of prostate health (General information)	prostate cancer<br>enlargement of the prostate gland<br>prostatitis<br>urinary infection	Screening	*No detail*	Aged between 50 and 75, or family history of prostate cancer
T16	Stomach Ulcer Test	Detect *Helicobacter pylori* antibodies.<br><br>(may cause chronic gastritis that can lead to stomach ulcer and/or cancer).	General Information<br><br>*No use statement*	*Helicobacter pylori* antibodies<br>*(no LOD stated)*	*No statement*	chronic gastritis<br>stomach ulcer<br>stomach cancer	Symptomatic diagnosis	frequent stomach pains<br>acid reflux	*No detail*
T17	Gluten Sensitivity Test	Detection of anti-tissue transglutaminase IgA antibodies associated with coeliac disease.<br><br>(indicates that it is likely that you have coeliac disease)	General Information<br><br>*No use statement*	Anti-tissue transglutaminase IgA antibodies<br>*(no LOD stated)*	*No statement*	gluten sensitivity<br>coeliac disease	Symptomatic diagnosis	chronic diarrhoea<br>abdominal pain<br>bloating<br>wind<br>weight loss<br>anaemia<br>osteoporosis<br>extreme fatigue<br>delayed growth in affected children	*No detail*
T18	One step Strep A Swab test	*No claim*	Opening paragraph<br><br>Early diagnosis	Group A streptococcal antigen<br>*(LOD stated)*	Group A streptococcal infection	acute pharyngitis<br>tonsilitis<br>impetigo<br>scarlet fever<br>rheumatic fever<br>glomerulonephritis	Symptomatic diagnosis	throat inflamed<br>tonsils inflamed	*No detail*
T19	Flowflex™ Influenza A/B Rapid Test (Self-Testing)	Detection of influenza virus type A and type B antigens	Intended Use statement<br><br>Screening test	Influenza virus type A and type B antigens<br>*(LOD stated)*	acute influenza type A and B viral infections	pneumonia<br>death	Symptomatic diagnosis<br>Screening	sudden onset of high fever<br>headache<br>muscle/joint pain	*No detail*

Test ID label	Test Product Name	Claim made on box	Indication for Use statement	IFU technical claim<br>*(Test threshold)*	IFU clinical claim	Outcomes associated with health condition being tested for	Implied Test Application (Screening, Diagnosis or Monitoring)	History or symptoms if for diagnosis	Population characteristics if for screening
			Aid in the diagnosis					fatigue<br>respiratory signs and symptoms<br>sore throat<br>cough (usually dry)<br>sputum<br>and so on.	
T20	Flowflex™ SARS-CoV-2 Antigen Rapid Test (Self-Testing)	Detection of SARS-CoV-2 nucleocapsid antigens	Intended Use statement<br><br>Suspected based on symptoms<br>Without symptoms	nucleocapsid antigen from SARS-CoV-2<br>*(LOD stated)*	COVID-19	*No detail*	Symptomatic diagnosis<br>Screening	first seven days of symptoms:<br>fever<br>fatigue<br>dry cough<br>nasal congestion<br>runny nose<br>sore throat<br>myalgia<br>diarrhea	without symptoms
T21	One step test for SARS-CoV-2 Antigen (Colloidal Gold)	*No claim*	Intended Use statement<br><br>Suspected based on symptoms<br>Without symptoms	SARS-CoV-2 antigen<br>*(LOD stated)*	SARS-CoV-2 infection	*No detail*	Symptomatic diagnosis<br>Screening	first seven days of symptoms:<br>headache<br>fever<br>cough<br>sore throat<br>loss of sense of taste or smell<br>shortness of breath<br>muscle pain	without symptoms
T22	STEPAHEAD® COVID-19 Antigen Rapid Test Kit (Swab) For Self-Testing	*No claim*	Intended use statement<br><br>*No use statement*	novel coronavirus SARS-CoV-2<br>*(LOD stated)*	SARS-CoV-2 infection	*No detail*	Symptomatic diagnosis<br>Screening	Known COVID-19	suspected Covid-19
T23	Microalbuminuria (MAU) Rapid Test Kit (Colloidal Gold)	*No clear claim*<br><br>(Detect increased level of albumin in urine as an indicator of chronic kidney disease)	Intended Use statement<br><br>Supplementary method for diagnosis	Levels of albuminuria<br>*(concentration more than 20 mg/L)*	diagnosis of chronic kidney injury (CKI)	glomerular disease<br>diabetic nephropathy<br>hypertension<br>cardiac insufficiency	Symptomatic diagnosis	*No detail*	*No detail*
T24	TSH Rapid Test Cassette	*No clear claim*<br><br>(Presence of TSH in human whole blood as an indicator of an underactive thyroid)	Intended Use statement<br><br>Preliminary diagnostic test<br>Screening	thyroid-stimulating hormone<br>*(concentration of 5μIU/mL)*	hypothyroidism (underactive thyroid)	*No detail*	Symptomatic diagnosis<br>Screening	Feeling tired<br>depressed<br>cold regularly<br>weight gain<br>dry skin<br>brittle hair<br>enduring constipation<br>menstrual cycle irregularities	*No detail*
T25	Ferritin Rapid Test Cassette	*No clear claim*<br><br>(Identifying iron deficiency and anaemia)	Intended Use statement<br><br>*No use statement*	Ferritin<br>*(less than 30ng/mL)*	Iron deficiency anaemia	heart failure<br>problems during pregnancy<br>delayed growth<br>development in children<br>hypothyroidism<br>vitamin C deficiency<br>coeliac disease<br>restless legs syndrome	Symptomatic diagnosis	extreme fatigue<br>difficulty concentrating<br>headache<br>pale skin<br>muscle and joint pain<br>weight gain<br>palpitations<br>sleep disturbances<br>weakness<br>chest pain<br>fast heartbeat<br>shortness of breath	*No detail*
T26	Iron Deficiency	*No clear claim*<br><br>(Detection of Ferritin in human fingerprick blood for iron deficiency anemia)	Opening paragraph<br><br>*No use statement*	Ferritin<br>*(less than 30ng/mL)*	Iron deficiency anaemia	hypothyroidism<br>vitamin C deficiency<br>coeliac disease<br>restless legs syndrome	Symptomatic diagnosis	paleness<br>feeling tired<br>headaches<br>faster heartbeat<br>shortness of breath during exercise	*No detail*
T27	Vitamin D Rapid Test Cassette	*No clear claim*<br><br>(Detect Vitamin D deficiency)	Intended Use statement<br><br>Preliminary diagnostic<br>Screening<br><br>FAQ section<br><br>Monitoring	Detection of 25-hydroxy Vitamin D<br>*(deficiency (0-10 ng/mL), (0-25 nmol/L)<br>insufficient (10-30 ng/mL), (25-75 nmol/L)<br>sufficient (30-100 ng/mL) (75-250 nmol/L))*	Vitamin D deficiency	rickets (children)<br>osteomalacia<br>postmenopausal osteoporosis<br>renal osteopathy<br>multiple sclerosis<br>cardiovascular diseases<br>pregnancy complications<br>diabetes<br>depression<br>strokes<br>autoimmune diseases	Symptomatic diagnosis<br>Screening<br>Monitoring	*No detail*	*No detail*

Test ID label	Test Product Name	Claim made on box	Indication for Use statement	IFU technical claim *(Test threshold)*	IFU clinical claim	Outcomes associated with health condition being tested for	Implied Test Application (Screening, Diagnosis or Monitoring)	History or symptoms if for diagnosis	Population characteristics if for screening
						flu<br>different cancers<br>infectious diseases<br>alzheimer<br>obesity<br>higher mortality etc			
T28	Vitamin D Test	Detects low levels of 25-hydroxy vitamin D in blood.<br><br>(an aid for screening for Vitamin Deficiency)	Intended Use statement<br><br>Preliminary diagnostic Screening<br><br>FAQ section<br><br>Monitoring	Detection of 25-hydroxy Vitamin D<br>*(deficiency (0-10 ng/mL), (0-25 nmol/L)*<br>*insufficient (10-30 ng/mL), (25-75 nmol/L)*<br>*sufficient (30-100 ng/mL) (75-250 nmol/L))*	Vitamin D deficiency	rickets (children)<br>osteomalacia<br>postmenopausal osteoporosis<br>renal osteopathy<br>multiple sclersosis<br>cardiovascular diseases<br>pregnancy complications<br>diabetes<br>depression<br>strokes<br>autoimmune diseases<br>flu<br>different cancers<br>infectious diseases<br>alzheimer<br>obesity<br>higher mortality etc	Symptomatic diagnosis<br>Screening<br>Monitoring	*No detail*	*No detail*
T29	Cholesterol Level Test	To check whether your overall cholesterol level is within normal limits.<br><br>(risk factor for hardening of the arteries (arteriosclerosis), stroke and cardiac arrest)	General Information<br><br>*No use statement*	Raised cholesterol level<br>*(above 5.2 mmol/L (200 mg/dL))*	Hypercholesterolemia	arteriosclerosis<br>stroke<br>cardiac arrest<br>circulatory problems<br>heart attack<br>health failure<br>coronary heart disease	Screening	*No detail*	*No detail*
T30	Blood Glucose Test	To check whether your overall blood glucose level is within normal limits.<br><br>(whether you are at risk of diabetes)	General Information<br><br>*No use statement*	Glucose levels<br>*(abnormal below 3.9mmol/L (70 mg/dL) or above 6.1 mmol/L (110 mg/dL))*	Blood sugar level	damage to blood vessels<br>impaired eyesight<br>kidney failure<br>nerve damage<br>erectile dysfunction<br>poor wound healing<br>cardiac arrest<br>strokes	Screening	*No detail*	*No detail*

AIDS – acquired immunodeficiency syndrome; CKI – chronic kidney infection; FOB – faecal occult blood; FSH – follicle-stimulating hormone; HIV – human immunodeficiency virus; IFU – Instructions for Use; LOD – limit of detection; MAU – microalbuminuria; PMSCs – progressive motile sperm cells; PSA – prostate specific antigen; STI – sexually transmitted infection; TSH – thyroid stimulating hormone.

Table A3: Details about test interpretation and recommendations following test results

Test ID label	Test Product Name	Biomarker	What indicates an abnormal result?	What action should you take?	What indicates a normal result?	What action should you take?	Concerns regarding instructions and interpretation
T1	Menopause Test	Follicle-stimulating hormone (FSH)	'If two purple lines appear on the result area, and the test line (lower line) is the same shade or darker than the control line (upper line) then your result is positive.'<br><br>'Only if both tests yield a positive result you can assume that you have reached post menopause.'	'You should repeat the test with the second test strip after exactly one week.'	'If two purple lines appear on the result area and the control line (upper line) is a significantly darker shade than the test line (lower line) or, if only the control line appears…you have not yet reached post menopause.'	'you do not need to use the second strip.'	• Results are classified as negative if either when the T line is clear or when the T line shows a line but a different shade of colour from the C line. (**HR**)<br>• Very similar shade of colour for both T and C line. (**HR**)
T2	Flourish Menopause Test Kit	Follicle-stimulating hormone (FSH)	'The result is positive if the FSH line is darker than the Control line. This result means that the FSH level is equal or higher than 25 mIU/mL.'<br><br>[after two abnormal tests] 'If the positive result is confirmed, you could be under menopausal process'<br>'the FSH level was elevated over an unusual period and could therefore indicate you are under menopausal process.'<br><br>'A positive test indicates that the FSH level is equal or higher than 25 mIU/mL which means that you are possibly under menopausal process.'	[after an abnormal first test] 'a second test must be performed 5 or 7 days later'<br>'a second test should be performed 5 or 7 days later.'<br><br>[after two abnormal tests] 'you are advised to consult your gynaecologist for a definitive diagnostic.'<br>'You should consult your gynaecologist to confirm or not the diagnosis of menopause.'	'The result is negative if the Control line is darker than the FSH line. This result means that the concentration of FSH is lower than 25 mIU/mL and that you are probably not under menopausal process.'	'if you feel signs of menopause as indicated above, it is recommended to perform another test 40 to 60 days later or consult your gynaecologist.'	• Results are classified as negative either when the T line is clear or when the T line shows a line but a different shade of colour from the C line. (**HR**)
T3	Menopause (FSH) Rapid Test	Follicle-stimulating hormone (FSH)	'POSITIVE: Two lines are visible and the line in test line region (T) is the same as or darker than the line in the control line region (C). A positive result means that the FSH level is higher than normal.'	NS	'NEGATIVE: Two lines are visible, but the line in the test line region (T) is lighter than the line in the control line region (C), or there is no line in the test line region (T). A negative result means that the FSH level is not elevated at this time.'	NS	• Instructions state: "Urine specimens may be stored at 2-8 °C for up to 48hr prior to testing". (LR)<br>• Instructions state: "For prolonged storage, specimens may be frozen and stored below -20°C". (LR)<br>• Instructions state: "Frozen specimens should be thawed and mixed before testing". (LR)<br>• The IFU does not give an interpretation of a negative test result in the subgroup who have menopausal symptoms. (**HR**)<br>• Negative results are not clear as they can both be classified as negative if either the T line is absent, or if the T line shows a line but a different shade of colour from the C line. (**HR**)
		Perimenopausal symptoms and irregular menstrual cycles	[after two abnormal tests] 'Most likely in perimenopause.'<br><br>[after one normal test and one abnormal test] 'May be in early stages of perimenopause'	[after two abnormal tests] 'Discuss methods and therapies to promote good health after menopause with your doctor. DO NOT immediately discontinue contraception.'<br><br>[after one normal test and one abnormal test] 'DO NOT immediately discontinue contraception.'	[after two normal tests] 'Most likely not experiencing perimenopause this cycle.'	[after two normal tests] 'If symptoms persist, repeat testing in the following month or review other possible causes for symptoms.'	
		Menopausal symptoms and no menstrual cycle for 12 months	[after one abnormal test] 'Menopause has most likely occurred.'	[after one abnormal test] 'Test may be repeated. Discuss methods and therapies to promote good health after menopause with your doctor.'	NS	NS	
T4	FSH Rapid Menopause Test Midstream	Follicle-stimulating hormone (FSH)	'POSITIVE: Two lines are visible and the line in test line region (T) is the same as or darker than the line in the control line region (C). A positive result means that the FSH level is higher than normal.'	NS	'NEGATIVE: Two lines are visible, but the line in the test line region (T) is lighter than the line in the control line region (C), or there is no line in the test line region (T). A negative result means that the FSH level is not elevated at this time.'	NS	• Instructions state: "Urine specimens exhibiting visible precipitates should be centrifuged, filtered or allowed to settle to obtain a clear specimen for testing". (MR)<br>• Instructions state: "Urine specimens may be stored at 2-8 °C for up to 48 prior to testing". (LR)<br>• The IFU does not give an interpretation of a negative test result in the subgroup who have menopausal symptoms. (**HR**)<br>• Negative results are not clear as they can both be classified as negative if either the T line is absent, or if the T line shows a line but a different shade of colour from the C line. (**HR**)
		Perimenopausal symptoms and irregular menstrual cycles	[after two abnormal tests] 'Most likely in perimenopause.'<br><br>[after one normal test and one abnormal test] 'May be in early stages of perimenopause'	[after two abnormal tests] 'Discuss methods and therapies to promote good health after menopause with your doctor. DO NOT immediately discontinue contraception.'<br><br>[after one normal test and one abnormal test] 'DO NOT immediately discontinue contraception.'	[after two normal tests] 'Most likely not experiencing perimenopause this cycle.'	[after two normal tests] 'If symptoms persist, repeat testing in the following month or review other possible causes for symptoms.'	
		Menopausal symptoms and no menstrual cycle for 12 months	[after one abnormal test] 'Menopause has most likely occurred.'	[after one abnormal test] 'Test may be repeated. Discuss methods and therapies to promote good health after menopause with your doctor.'	NS	NS	
T5	SP-10 Male Fertility Rapid Test	Acrosomal protein SP-10	'ABNORMAL. One coloured line appears in the control line region (C). No line appears in the test line region (T).'<br><br>'Fresh samples collected are generally liquefied within 60 minutes, and if they do not liquefy within 60 minutes it means an abnormal result.'<br>'An absence of the test line indicates that the sperm concentration is less than 15 million/mL.'	'Sperm concentration is one of several semen analysis tests. There are other factors that should be considered, including motility. Therefore, it is strongly recommended that you seek expert medical advice if you get an abnormal result.'<br><br>'It would be advisable to see your medical professional who can advise what can be done to improve the sperm concentration.'	'NORMAL: Two coloured lines appear. One coloured line should be in the control line region (C) and another apparent coloured line should be in the test line region (T).<br>NOTE: The intensity of the colour in the test line region will vary depending on the concentration of SP-10 protein present in the specimen. Therefore, any shade of colour in the test line region (T) should be considered normal.'<br><br>'The Male Fertility Test (SP-10 male fertility rapid test cassette) detects SP-10, and gives a positive result, when sperm concentration is above 15 million/ml in semen - a level internationally accepted as the minimum level of sperms for normal fertility.'	NS	
T6	SpermCheck Fertility	NS	'look at the Test Line position in the result window..If you do not see a line at the T position, your sperm count is less than 15 million per millilitre.'	'you should consult your doctor for a complete fertility evaluation.'	'The Test Line may or may not be as dark as the Control Line. If you see any line at all at the Test (T) position and the Control (C) position, your test result is positive, no	'if you and your partner have been trying to start a family without success for a year or more, you should see a doctor for a complete semen analysis and to discuss	• IFU labels the lateral flow test as positive as normal, whereas the negative as low. (LR)

Test ID label	Test Product Name	Biomarker	What indicates an abnormal result?	What action should you take?	What indicates a normal result?	What action should you take?	Concerns regarding instructions and interpretation
			'a negative SpermCheck Fertility test result alone does not prove that you are infertile. About 5% of fertile men have sperm counts below 15 million per millilitre, so you may still be able to father a child naturally'	'you should have a complete semen analysis and talk to a doctor about possible treatments for sub-fertility, especially if you and your partner have been trying to start a family without success.'	matter how faint the line is or how the Test Line compares to the Control Line.'<br><br>'look at the Test Line position in the result window...If you see a reddish line here, your sperm count is at least 15 million per millilitre.'<br><br>'a positive SpermCheck Fertility test result alone does not prove that you are fertile since there are several other factors that can influence a man's ability to father a child.'	treatments that could benefit you, even if your SpermCheck test result is positive.'<br><br>'If you and your partner are unable to conceive a child after several months of trying, you should both have full fertility evaluations, even if your SpermCheck test result was positive.'	
T7	SwimCount™ Sperm Quality Test	Concentration of Progressive Motile Sperm Cells (million/mL)	'Your concentration of PMSCs/mL is below the normal level for fertile men according to the World Health Organisation (WHO). Men with a LOW concentration of PMSCs/mL have been shown to have a lower chance of making their partner pregnant in a natural way compared to men with a normal concentration of PMSCs/mL.'<br><br>'A LOW result means that the concentration of the PMSCs/mL in your semen sample is lower than for fertile men. A LOW result does not necessarily mean that you will be unable to make a woman pregnant in a natural way as sperm quality varies over time.'	'Lifestyle changes may improve the concentration of PMSCs/mL which you can check with further SwimCount tests or consult your doctor.'<br><br>'If you want to see a possible change in your sperm quality after lifestyle changes, you should wait at least 3 months before testing again.'<br><br>'we recommend that you change your lifestyle, wait for 3 months and take another SwimCount Test or consult your doctor for further fertility testing.'	'Your concentration of PMSCs/mL is at the NORMAL (Mid) level for fertile men according to the World Health Organisation (WHO). Men with a NORMAL (Mid) concentration of PMSCs/mL have been shown to have a good chance of making their partner pregnant in a natural way within 12 months.'<br><br>'Your concentration of PMSCs/mL is at the NORMAL (High) level for fertile men according to the World Health Organisation (WHO). Men with a NORMAL (High) concentration of PMSCs/mL have been shown to have a high chance of making their partner pregnant in a natural way within 12 months.'<br><br>'The darker the colour the more Progressive Motile Sperm Cells/mL are present in the semen sample.'	'One in six couples have difficulties getting pregnant and there are factors other than the number of PMSCs/mL that may affect male fertility. These factors are not assessed with this test. If you and your partner have been trying to get pregnant for 12 months without success, you should see your doctor for further testing and investigation'.	• The shades of blue used to indicate two of the three levels of concentration are similar. (MR)<br>• Within the IFU, the definitions of sensitivity and specificity metrics have been interpreted as predictive values. This means that the post-test probability of having a normal sperm count (PPV), given a normal result, is underestimated by 2.6 percentage points (98.6% vs 96% sensitivity), but this also means that the post-test probability of having an abnormal sperm count (NPV), given an abnormal result, is overestimated by 15.2 percentage points (75.8% vs 91% specificity). (LR)
T8	SURE CHECK® HIV Self-Test	Antibodies to HIV-1 and HIV-2	'REACTIVE SELF-TEST<br>If your self-test looks like one of the examples below, the result is positive.<br>2 lines appear: the control line and the test line. Either line may be lighter or darker than the other.<br>YOUR TEST IS REACTIVE YOU ARE PROBABLY HIV-POSITIVE.'	'CONSULT A DOCTOR, as soon as possible, and inform him/her that you have just done a self-test for HIV and that your result was positive.'<br><br>'Your self-test must be verified by having a confirmatory lab test.'<br><br>'PROTECT YOURSELF AND OTHERS'<br><br>'Avoid any activity that could transmit HIV to others until you have received the results of your confirmatory test.'<br><br>'If your result if positive, you should not take any decision about your condition without first consulting a healthcare professional who will confirm your result.'	'NON-REACTIVE SELF-TEST<br>If your self-test looks like the example below, the result is negative.<br>1 line appears: the control line. This line may be light or dark.<br>YOUR TEST IS NON-REACTIVE YOU ARE PROBABLY HIV-NEGATIVE.'<br><br>'If you think you may have been exposed to HIV in the last 3 months, you cannot be certain about being HIV-negative at this time.'	'If your result is negative, it is important to ensure that you are not in the window period (seroconversion) and to consult a doctor.'<br><br>[if there are concerns about recent HIV exposure] 'You will need to redo the self-test once 3 months have passed since your most recent risk exposure to HIV.'<br><br>'You should use caution if your negative result is before the 12 weeks from the last possible exposure to the virus.'	
T9	Female Chlamydia STI Test Kit	NS	'The result is considered positive if two purple lines appear in the results window (at 'C' and 'T'), even if the line at 'T' is very faint.'	'you should see your doctor or other appropriate healthcare professional to discuss the result. Please take these instructions with you to your appointment so that they can have a clearer picture of the test that you have performed.'<br><br>'In the event of an infection, it is recommended that your sexual partner also takes a test and undergoes treatment.'	'The result is considered negative if there is only one purple line at 'C'.'<br><br>'you are highly unlikely to have a Chlamydia trachomatis infection'	'it is still recommended that you consider testing regularly.'	
T10	Women's Intimate Self-test	Vaginal pH	'If your test result is: [brown or blue]<br>Your vaginal pH is higher than normal (pH > 4.4)<br><br>And you also have these symptoms:<br>Itching, Unpleasant fishy smell, Abnormal liquid white to greyish discharge<br><br>You probably have:<br>Bacterial infection or vaginosis<br><br>And you also have these symptoms:<br>Itching, Pain when urinating, Unpleasant fishy smell, Abnormal foamy yellow to greenish discharge<br><br>You probably have:<br>Trichomoniasis infection'	[results suggestive of bacterial infection or vaginosis] 'Ask your pharmacist for advice about suitable products to treat this infection. If problem persists, consult a doctor.'<br><br>[results suggestive of trichomoniasis infection] 'Consult your doctor for advice on treatment'	'If your test result is: [yellow]<br>Your vaginal pH is normal<br><br>And you also have these symptoms:<br>Itching, Burning, Abnormal white odourless discharge (like cottage cheese)<br><br>You probably have:<br>Thrush'	[results suggestive of thrush] 'Ask your pharmacist for advice about suitable products to treat this infection. If problem persists, consult a doctor.'<br><br>'If you are concerned about your vaginal health even though the pH value is normal, consult your doctor.'	• The differences between pH 4.4 (marked as "normal") and 4.7 to 5 (marked as "higher than normal") are not easy to distinguish. (MR)

Test ID label	Test Product Name	Biomarker	What indicates an abnormal result?	What action should you take?	What indicates a normal result?	What action should you take?	Concerns regarding instructions and interpretation
T11	Canestest Self-test for Vaginal Infections	Vaginal pH	'A positive test (colour change to blue/green) indicates a higher vaginal pH level.'<br><br>'Colour change to blue or green (positive result):<br><br>If the tip is stained blue or green after 10 seconds, this indicates your vaginal pH is increased. If you are also suffering of the following symptoms:<br><br>- Thin milky (consistent), white-grey discharge with a fishy odour, it may indicate bacterial vaginosis (BV) infection.<br><br>- Yellow-green, frothy discharge associated with bad odour and painful urination, it may indicate trichomoniasis infection.<br><br>Any partial blue or green stain on the yellow indicator swab tip should be considered as a positive result.'	'In case of doubts, please consult your doctor or healthcare professional for advice. For example, tip is stained blue or green but you do not have the associated symptoms described in the table, please consult your doctor or healthcare professional.'<br><br>[results suggestive of bacterial vaginosis] 'Consult your pharmacist, doctor or healthcare professional, for advice on treatment'<br><br>[results suggestive of trichomoniasis] 'Consult your doctor for prescription'	'A negative test (no colour change) means a normal vaginal pH.'<br><br>'No colour change/remains yellow (negative result):<br><br>If the tip is not stained blue or green after 10 seconds this indicates your vaginal pH is normal. The probability of having a bacterial or trichomonas infection is low.<br><br>However, if you are suffering from the following symptoms:<br>- White, thick, cottage cheese-like discharge without offensive odour, it may indicate vaginal yeast infection, known as thrush.'	[results suggestive of thrush] 'Consult your pharmacist doctor or healthcare professional and consider using over the counter thrush products.'<br><br>[negative test and no symptoms, options for treatment] 'None'	
T12	Urine Infection Test	Protein, nitrite and leucocytes	'If the colour of the test strip has changed to purple, then leucocytes have been found in your urine (positive result for leucocytes).<br><br>If the colour of the test field has changed to pink, then nitrite has been found in your urine (positive result for nitrite).<br><br>If the colour of the test strip has changed to green, then protein has been found in your urine (positive result for protein).'<br><br>'Remember that a positive result does not mean that all three substances have been detected in your urine. Even if your result is positive for just one of them, it is most likely that something is wrong with your urine even if the reason may not be a urinary infection.'	'If any one or more of the three substances are detected then the user should consult their GP so that further investigations can be carried out.'<br><br>'Get in touch promptly with your own doctor, who will be able to give a more accurate diagnosis. When you visit your doctor, please take these instructions with you so that he/she will be better informed as to the type of test that you have performed.'<br><br>'Your own doctor will diagnose your specific condition if protein is found in your urine, determining why it has been detected.'	'If the colour of the test field has not changed or remained yellow, then no leucocytes have been detected in your urine (negative result for leucocytes).<br><br>If the colour of the test field has not changed or remained white, then no nitrite has been detected in your urine (negative result for nitrite).<br><br>If the colour of the test field has not changed or remained yellow, then no protein has been detected in your urine (negative result for protein).'<br><br>'Remember that your test result is only negative if the result on the test field for all three substances is negative.'	'if you still feel the signs and symptoms of a UTI or have any other symptoms, then contact your own doctor to arrange a more thorough examination.'	• Very similar shade of colour on dipstick. (**HR**)
T13	Bowel Health Test	Human haemoglobin	'The test is positive, if within the reaction time of five minutes, two purple lines appear in the result window of the test cassette at both "C" and "T", even the line shown at "T" is very faint This means that blood has been detected in your stool.'	'You should visit your doctor and ask for a more detailed medical examination to be undertaken.'<br><br>'Discuss the result with your doctor.'<br><br>'It might be helpful to take these instructions with you to show your doctor, so you can give him or her a better idea of the type of test carried out.'	'The test is negative if only one purple line appears in the result window at "C". This means that no blood was detected in your stool sample.'<br><br>'A negative result does not entirely exclude the possibility of a bowel condition, since some colon tumours only bleed intermittently. Around 25% of tumours do not bleed constantly.'	'It is important to test yourself or arrange to be tested annually from age 40 at the latest in order to keep your risk as low possible.'	• There is no threshold stated on the test to compare with the NHS screening committee FIT test of 120 µg Hb/g.[1] (**HR**)
T14	FOB Rapid Test (Faeces)	Human haemoglobin	'POSITIVE: Two coloured lines appear. One coloured line should be in the control line region (C) and another apparent coloured line should be in the test line region (T).<br><br>NOTE: The intensity of the colour in the tets line region (T) will vary depending on the concentration of fecal occult blood present in the specimen. Therefore, any shade of colour in the test line region (T) should be considered positive.'	'You should visit your doctor for advice.'	'NEGATIVE: One coloured line appears in the control line region (C). No line appears in the test line region (T).'	NS	• The claimed threshold of 6 µg Hb/g is lower than the threshold used in the National Screening Committee FIT test of 120 µg Hb/g.[1] (**HR**)<br>• The instructions will not be readable during the sample process as they form part of the collection paper which a user tapes onto their toilet seat where they collect the faecal sample. (**HR**)
T15	Prostate Health Test	Prostate specific antigen (PSA)	'Positive result<br>Two coloured lines appear in the window under the marks T (test) and C (control). The T line may be much lighter or darker in colour than the C line. Two lines mean that your PSA level is higher than normal'<br><br>'If the result is positive, it means that the PSA level in your blood is higher than normal (4ng/ml).'<br><br>'Levels above normal may indicate a problem such as an enlarged prostate, prostatitis or the development of prostate cancer.'<br><br>'many men with a raised PSA (≥4.0ng/ml) may not have prostate cancer'	'you should consult your doctor.'<br><br>'you should consult your doctor and mention your test results.'	'Negative result<br>Only one coloured line appears under the C mark (control). This means that your PSA level is in the normal range.'<br><br>'If the result is negative it means that your PSA level is in the normal range (less than 4ng/ml).'	'If you are between 50 and 75 years old, or have a family history of prostate cancer, it is recommended to test regularly.'	• No reference ranges provided with which to interpret results. Continuous measurements of are needed to interpret this biomarker,[2] which cannot be done using a binary lateral flow test. (**HR**)
T16	Stomach Ulcer Test	Helicobacter pylori antibodies	'Positive result<br>Two coloured lines appear in the window under the marks T (Test) and C (Control). The T line may be much lighter or	'you should consult your doctor.'<br><br>'you should speak to your doctor and mention the	'Negative result<br>Only one coloured line appears under the mark C (Control). This result means that no H. pylori antibodies were detected in your blood sample.'	'you should speak to your doctor if your symptoms continue.'	

Test ID label	Test Product Name	Biomarker	What indicates an abnormal result?	What action should you take?	What indicates a normal result?	What action should you take?	Concerns regarding instructions and interpretation
			darker in colour than the C line. Two lines mean that H. pylori antibodies have been detected in your blood'<br><br>'The test result should be considered positive even if the test line (T) is very faint.'<br><br>'Positive results may also be detected if you have been recently treated for a H. pylori infection.'<br><br>'the presence of H. pylori antibodies could be due to a previous infection that you may have been treated for.'	test results. Your doctor will decide whether any additional investigation should be performed'			
T17	Gluten Sensitivity Test	Anti-tissue transglutaminase IgA antibodies	'Positive result<br>Two coloured lines appear in the window under the marks T (Test) and C (Control). The T line may be much lighter or darker in colour than the C line. Two lines means that the blood sample contains anti-t-TG IgA type antibodies'<br><br>'The test result should be considered positive whatever the colour intensity of the test line (T) is.'	'you should consult a doctor.'<br><br>'You should speak to your doctor and mention the test results. Your doctor will decide whether any additional investigation should be performed.'	'Negative result<br>Only one coloured line appears under the mark C (Control). This means that no anti-t-TG IgA type antibodies have been detected in the blood sample.'<br><br>'in limited cases you may also get a negative result if you are IgA deficient'	'You should speak to your doctor if your symptoms continue.'	• No reference ranges provided with which to interpret results. Continuous measurements of are needed to interpret this biomarker,[3] which cannot be done using a binary lateral flow test. (**HR**)
T18	One step Strep A Swab test	Group A streptococcal antigen	'Positive (+)<br>If two colour bands are visible both in the test (T) band, the result is positive. It indicates that you may be in a stage of Strep A infection'<br><br>This test does not provide any further information about pharyngitis other than the possibility of Strep A infection.'<br><br>'A positive test indicates you may be with group A streptococcal infection.'<br><br>'When the group A streptococcal antigen levels in specimens are at or above the target cutoff, there is a visible coloured band in the test region and indicates a positive result.'	'Please see a doctor for medical aid.'	'Negative (-)<br>If only one coloured band appears in the control (C) region, no color bad appears in the test region, the result is negative. It indicates that the concentration of the group A streptococcal antigen is zero or below the detection limit of the test.'<br><br>'a negative test result means that you are probably not infected'<br><br>'Absence of this colored band in the Test region (T) suggests a negative result'	'If symptoms persist or intensify, you should consult with your doctor.'<br><br>'if you are serious complication, including rheumatic fever and inflammation you should consult with your doctor.'	
T19	Flowflex™ Influenza A/B Rapid Test (Self-Testing)	Influenza A antigen and influenza B antigen	'Two distinct colored lines appear. One line in the control line region (C) and the other line in the test line region (B). This means that the presence of Influenza B antigen was detected.<br><br>'Two distinct colored lines appear. One line in the control line region (C) and the other line in the test line region (A). This means that the presence of Influenza A antigen was detected.'<br><br>'Three distinct colored lines appear. One line in the control line region (C) and one line in the test line region (B) and one line in the test line region (A). This means that the presence of influenza A antigen and Influenza B antigen were both detected.'<br><br>'A positive test result does not rule out co-infections with other pathogens.'	NS	'Only one colored control line appears in the control region (C). No apparent colored line appears in the test line region (A/B). This means that no Influenza B or Influenza A antigen was detected.'<br><br>'A negative test is presumptive and Negative results do not preclude influenza virus infections'<br><br>'A negative test result is not intended to rule out other viral or bacterial infections.'	[a negative test] 'should not be used as the sole basis for treatment or other patient management decisions.'	
T20	Flowflex™ SARS-CoV-2 Antigen Rapid Test (Self-Testing)	SARS-CoV-2 nucleocapsid antigen	'Both the control line (C) and test line (T) appears. This means that SARS-CoV-2 antigen was detected. NOTE: Any faint line in the test line region (T) should be considered positive.<br>A positive test means that it is very likely you currently have COVID-19 disease.'<br><br>'Positive results indicate the presence of viral antigens'<br><br>'A positive test result does not differentiate between SARS-CoV and SARS-CoV-2.'<br><br>'A positive test result does not rule out other viral or bacterial infections.'<br><br>'Positive results do not rule out bacterial infection or co-infection with other viruses. The agent detected may not be the exact cause of disease.'	'Contact your doctor / general practitioner or the local health department immediately. Follow the local guidelines for self- isolation. A PCR confirmation test should be carried out.'<br><br>'individual history and other diagnostic information is necessary to determine infection status.'	'Only the control line (C) and no test line (T) appears. This means that no SARS-CoV-2 antigen was detected. A negative test result indicates that you are unlikely to currently have COVID-19 disease.;'<br><br>'Negative results do not rule out SARS-COV-2 infection.'<br><br>'A negative test result does not rule out other viral or bacterial infections.'<br><br>'Negative results from individuals with symptoms beyond seven days should be treated as likely negative.'<br><br>'There may be an infection even if the test is negative.'	'Continue to follow all applicable rules and protective measures when contacting with others.'<br><br>[if infection still suspected] 'repeat the test after 1 – 2 days, as the coronavirus cannot be precisely detected in all phases of an infection.'<br><br>'Confirm with a molecular assay, if necessary.'	
T21	One step test for SARS-CoV-2 Antigen (Colloidal Gold)	SARS-COV-2 antigen	'Positive (+):	'individual history and other diagnostic information is necessary for determine infection status.'	'Negative (-):<br>Only the control line (C) and no test line (T) appear, this indicates no SARS-CoV-2 antigen was detected.	'If necessary, it should be confirmed with a molecular assay.'	

Test ID label	Test Product Name	Biomarker	What indicates an abnormal result?	What action should you take?	What indicates a normal result?	What action should you take?	Concerns regarding instructions and interpretation
			Both the control line (C) and tets line (T) appear, this indicates the presence of SARS-CoV-2 antigen. Any faint line in the test line (T) should be considered positive.<br>Note: Positive results indicate the very likely infected COVID-19.'<br><br>'Positive results indicate the presence of SARS-CoV-2 antigen'	'Contact your doctor or the local health department immediately. Follow the local guidelines for self-isolation and confirmed by a molecular testing method.'	Note: Negative results indicate the unlikely infected COVID-19.'<br><br>'There may be an infection even if the test is negative.'<br><br>'Negative results do not rule out SARS-CoV-2 infection. Negative results for individuals with symptoms similar to COVID-19 infection for more than seven days should be treated as negative possibly.'	'Continue to follow all applicable rules and protective measures when contacting with others.'<br><br>[if infection still suspected] 'repeat the test after 1 - 2 days or confirm it by a molecular testing method.'	
T22	STEPAHEAD® COVID-19 Antigen Rapid Test Kit (Swab) For Self-Testing	SARS-CoV-2 nucleocapsid protein antigen	'POSITIVE RESULT<br>A colored line appears in the control line region (C) and a colored line appears in the test line region (T).<br>NOTE: The intensity of the color in the test line region will vary dependent on the concentration of COVID-19 antigen in the specimen. Therefore, any shade of color in the test line region should be considered positive.'<br><br>'Positive results are indicative of the presence of SARS-CoV-2.'<br><br>'Positive results do not rule out bacterial infection or co-infection with other viruses.'<br><br>'there is currently a suspicion of a COVID-19 infection.'<br><br>'The colour intensity of the positive line shall not be evaluated as quantitative or semi-quantitative.'	'Individuals who test positive should self-isolate and seek additional care from their healthcare provider.'<br><br>'contact your doctor / general practitioner or the local health department immediately<br>Comply with local guidelines for self-isolation to have a PCR confirmatory test performed'	'Negative results do not preclude SARS-CoV-2 infection.'<br><br>'NEGATIVE RESULT:<br>A colored line appears in the control region (C) and no line appears in the test line region (T).'<br><br>'An infection may also be present if the test is negative'<br><br>'A negative result does not exclude the possibility of COVID-19 infection.'	'Individuals who test negative and continue to experience COVID-like symptoms should seek follow up care from their healthcare provider.'<br><br>'Continue to comply with all applicable rules regarding contact with others and protective measures<br><br>[if an infection is still suspected] ' repeat the test after 1 - 2 days, as the coronavirus cannot be accurately detected in all phases of an infection'	
T23	Microalbuminuria (MAU) Rapid Test Kit (Colloidal Gold)	Albuminuria	'Positive<br>One coloured line appears in the control line region (C). No line appears in the test line region (T). A positive result with the test indicates the albumin in urine is more than the cut off value.<br>The result should be considered positive even it there is a faint line in the T line region, like G3-G4 shown in the colour card included. It indicates the concentration of albumin present in the urine is close to the cutoff value.'<br><br>'A positive result with the test indicates the albumin concentration in urine is more than 20mg/L, and does not necessarily indicate kidney injury.'	NS	'Negative (-)<br>Two red lines are visible. One is located in the test line region (T), the other is in control line region A negative result indicates the albumin in urine is less than the cut off value.'	NS	• The threshold concentration is 20 mg/L (usually mg/g). A reference range of 30 mg/g or less is normal; this is likely to over-diagnose. (**HR**)<br>• Instructions state: "If there is sediment at the bottom of the container, please centrifuge or filter". (MR)<br>• Instructions state: "urine specimen can be refrigerated at 2-8 C for 48 hours". (LR)<br>• Instructions state: "For long term storage, specimens should be kept below -20 C". (LR)<br>• Instructions state "Specimens may be infectious or be a potential biological hazard. When collecting another individual urine, wear Disposable gloves and masks to prevent contact". (LR)<br>• A colour card is used to decide whether a value is negative or positive, which has 11 values, with very subtle differences between them. (**HR**)<br>• Instructions state: "results are considered positive even if there is a faint line in the T line region, like G3-G4 shown in the colour card included". There are many "faint" lines which makes this assessment ambiguous. (**HR**)<br>• The IFU is confusing regarding whether a positive result is abnormal or normal. Contrary to normal usage (of lateral flow tests), high levels of albuminuria, which is abnormal, show no T line but only a C line. Lower levels of albuminuria, which is normal, show a T line and a C line. (**HR**)<br>• Contrary to normal usage (of lateral flow tests), faint T lines like G3-G4 marked on the colour chart are classified as positive (alongside the test when no T line is seen). (**HR**)
T24	TSH Rapid Test Cassette	Thyroid-stimulating hormone (TSH)	'POSITIVE: Two coloured lines appear. Both T (test) and C (control) line appear.<br>This result means that the TSH level is higher than the normal (5µIU/mL)'<br><br>'The test should be considered positive whatever the color intensity of the test line is.'<br><br>'a TSH level over 5µIU/mL in the case of a positive result, indicates an under active thyroid (hypothyroidism)'	'repeatedly abnormal results should be discussed with a doctor or medical professional'<br><br>'you should consult a physician.'<br><br>'the physician will decide which additional analysis should be performed.'<br><br>'A positive test must be confirmed using a quantitative laboratory TSH assay.'	'NEGATIVE: One coloured line appears. Only control line appears (C).<br>This result means that the TSH level is not in the range to consider hypothyroidism.'<br><br>'If the result is negative, it means the TSH level is below 5µIU/mL and is within the normal range. Although rare, hyperthyroidism cannot be excluded based on such test results.'	[hyperthyroidism cannot be excluded so] 'If the symptoms persist, it is recommended to consult a physician.'	• In the text and figures, steps 6 and 7 are in the opposite order, stating to add buffer first in the figure and to add blood first in the text. These instructions are different in the IFU of the same test marketed by a different distributor. (**HR**)<br>• The text instructions state to use 2 drops of blood, whereas the pipette could hold more blood than this. These instructions are different in the IFU from a different distributor which directs use of the whole pipette full of blood. (**HR**)

Test ID label	Test Product Name	Biomarker	What indicates an abnormal result?	What action should you take?	What indicates a normal result?	What action should you take?	Concerns regarding instructions and interpretation
							• No reference ranges provided with which to interpret results. Continuous measurements are needed to interpret this biomarker,[4] which cannot be done using a binary lateral flow test. (**HR**)
T25	Ferritin Rapid Test Cassette	Ferritin	'Abnormal: One coloured line appears. Only C (Control) line appears.<br>This result means that the ferritin concentration in blood is too low.'<br><br>'An abnormal result means that the ferritin concentration in the blood is lower than 30ng/mL and a possible iron deficiency.'<br><br>'Abnormal results can be obtained even in cases of no iron deficiency situation.'<br><br>'it may be an iron deficiency'	'repeatedly abnormal results should be discussed with a doctor or medical professional.'<br><br>'you should consult the physician and show the test result to him/her. Then, the physician will decide whether additional analysis should be performed.'	'Normal: Two coloured lines appear. Both T (Test) and C (Control) line appear. This result means that the Ferritin concentration in blood is normal and that there is no potential iron deficiency.'<br><br>'If the result is normal, it means that the ferritin level is higher than 30 ng/mL and is within the normal range.'	'if the symptoms persist, it is recommended to consult a physician.'	• No reference ranges provided with which to interpret results. Continuous measurements are needed to interpret this biomarker,[5,6] which cannot be done in a binary lateral flow. (**HR**)<br>• The IFU states that pregnant women should be tested which is likely to lead to overdiagnosis and anxiety as the reference range in pregnant women is very different. (**HR**)
T26	Iron Deficiency	Ferritin	'Abnormal: Only one line appears. Only control line (C) appears.<br>This result means that the ferritin concentration in blood is too low.'<br><br>'An abnormal result means that the ferritin concentration in the blood is lower than 30ng/mL and a possible iron deficiency.'<br><br>'Abnormal results can be obtained even in cases of no iron deficiency situation.'<br><br>'it may be an iron deficiency'	'repeatedly abnormal results should be discussed with a doctor or medical professional.'<br><br>'you should consult the physician, the physician will decide whether additional analysis should be performed.'	'Normal: Two lines appear. Both T (Test) and C (Control) lines appear. This result means that the ferritin concentration in blood is normal and that there is no potential iron deficiency.'<br><br>'If the result is normal, it means that the ferritin level is higher than 30 ng/mL and is within the normal range.'	'if the symptoms persist, it is recommended to consult a physician.'	• The numbers on the text instructions do not agree with the illustration. (**HR**)<br>• No reference ranges provided with which to interpret results. Continuous measurements are needed to interpret this biomarker,[5,6] which cannot be done using a binary lateral flow test. (**HR**)<br>• The IFU states that pregnant women should be tested which is likely to lead to overdiagnosis and anxiety as the reference range in pregnant women is very different. (**HR**)
T27	Vitamin D Rapid Test Cassette	25-hydroxy Vitamin D	'Two coloured lines appear. One line in the control region (C) and another should be in the test line region (T).<br><br>Deficient.<br>The line intensity in the test region (T) is equal to or darker than 10ng/mL line depicted on the colour card provided with the kit.<br><br>Insufficient.<br>The line intensity in the test region (T) is darker than the 30 ng/mL line depicted on the colour card provided with the kit and lighter than 10 ng/mL line depicted on colour card provided with the kit.'<br><br>'Excess.<br>One coloured line appears in the control line (C). No apparent coloured line appears in the test line (T).'<br><br>'If the result is deficient or insufficient, it means that the vitamin D level in blood is less than 30ng/mL'	[after a deficient or insufficient result] 'Vitamin D supplements can be recommended'<br>'you should consult a physician to show the test result. Then, the physician will decide whether additional analysis should be performed.'<br><br>'If the result is excess, it is recommended to consult physician.'	'Sufficient.<br>Two coloured lines appear, one line should always be in the control region (C) and a faint colored line appears in the test region (T). The line intensity in region (T) is equal to or lighter than 30 ng/mL line depicted on Color card.'<br><br>'If the result is sufficient, it means that the vitamin D level is higher than or equal to 30ng /mL and is within the normal range.'<br><br>'A case of vitamin D toxicity (hypercalcemia), though rare, but cannot be excluded based on such test results.'	'if symptoms persist, it is recommended to consult a physician.'	• No reference ranges provided with which to interpret results. Continuous measurements of are needed to interpret this biomarker,[7] which cannot be done in a binary lateral flow. (**HR**)<br>• Interpretation of the abnormal results is confusing as either the presence or absence of the T line could represent an abnormal result. There are three categories which give abnormal results, "deficient", "insufficient" and "excess", with the former two indicated by the presence of a darker T line and the latter indicated by the absence of a T line. (**HR**)<br>• The insufficient section is wrongly marked as white in the colour card. This is different to the colour card from a different distributor of the same test. (**HR**)<br>• The differences in shade of pink between insufficient and sufficient are very subtle. (**HR**)
T28	Vitamin D Test	25-hydroxy Vitamin D	'Two coloured lines appear. One line in the control region (C) and another should be in the test line region (T).<br><br>Deficient.<br>The line intensity in the test region (T) is equal to or darker than 10ng/mL line depicted on the colour card provided with the kit.<br><br>Insufficient.<br>The line intensity in the test region (T) is darker than the 30 ng/mL line depicted on the colour card provided with the kit and lighter than 10 ng/mL line depicted on colour card provided with the kit.'<br><br>'Excess.<br>One coloured line appears in the control line (C). No apparent coloured line appears in the test line (T).'<br><br>'If the result is deficient or insufficient, it means that the vitamin D level in blood is less than 30ng/mL'	[after a deficient or insufficient result] 'Vitamin D supplements can be recommended'<br>'you should consult a physician to show the test result. Then, the physician will decide whether additional analysis should be performed.'<br><br>'If the result is excess, it is recommended to consult physician.'	'Sufficient.<br>Two coloured lines appear, one line should always be in the control region (C) and a faint colored line appears in the test region (T). The line intensity in region (T) is equal to or lighter than 30 ng/mL line depicted on Color card.'<br><br>'If the result is sufficient, it means that the vitamin D level is higher than or equal to 30ng /mL and is within the normal range.'<br><br>'A case of vitamin D toxicity (hypercalcemia), though rare, but cannot be excluded based on such test results.'	'if the symptoms persist, it is recommended to consult a physician.'	• No reference ranges provided with which to interpret results. Continuous measurements of are needed to interpret this biomarker,[7] which cannot be done in a binary lateral flow. (**HR**)<br>• Interpretation of the abnormal results is confusing as either the presence or absence of the T line could represent an abnormal result. There are three categories which give abnormal results, "deficient", "insufficient" and "excess", with the former two indicated by the presence of a darker T line and the latter indicated by the absence of a T line. (**HR**)<br>• The insufficient section is wrongly marked as white in the colour card. This is different to the colour card from a different distributor of the same test. (**HR**)<br>• The differences in shade of pink between insufficient and sufficient are very subtle. (**HR**)

Test ID label	Test Product Name	Biomarker	What indicates an abnormal result?	What action should you take?	What indicates a normal result?	What action should you take?	Concerns regarding instructions and interpretation
T29	Cholesterol Level Test	Total cholesterol	'High Cholesterol<br>High levels are values above 5.2 mmol/L. Your cholesterol level is most probably high if your reading shows values above 5.2 mmol/L.<br><br>This is also the case if you have had difficulty deciding between the 200 mg/dL and the 225 mg/dL colour range.'<br><br>'If your cholesterol level is higher than the indicated normal range you should take the result seriously, although a one-off raised level by itself is not particularly meaningful.'	'If a high level of cholesterol is detected, you should speak to your doctor who will provide further advice and treatment on the reduction and control of cholesterol levels.'<br><br>'If your level appears high, contact your doctor for further tests.'<br><br>'If your cholesterol level appears high, you should see your doctor, who will repeat the test using another method which may involve distinguishing between HDL (high-density cholesterol) and LDL (low-density cholesterol) as well as the triglyceride levels in your blood, in order to better assess your risk of arteriosclerosis. It might be helpful to take these instructions for use with you to give your doctor in order to give a better idea of the type of test you have performed yourself.'	'Normal Cholesterol<br>Normal readings for adults are up to 5.2 mmol/L (200mg/dL). Your cholesterol level is most probably in this range if your reading shows values up to 5.2mmol/L.'	NS	• The results can give a mottled pattern without a clear single value. (MR)
T30	Blood Glucose Test	Blood glucose	'Outside Normal Range<br>If your test result is clearly below 3.9mmol/L (70 mg/dl) or above 6.1 mmol/L (110mg/dl) your blood sugar level is outside the normal range.'	'Repeat using the second test on a different day. If your reading is again outside of the normal range you should discuss this with your doctor.'<br><br>'If after using both tests, your blood sugar level on an empty stomach is significantly above or below the normal range of between 3.9mmol/L (70 mg/dl) and 6.1 mmol/L (110mg/dl), you should contact your doctor for a detailed assessment. It might be helpful to take these instructions with you to show your doctor and give them a better idea of the type of test you have carried out.'	'Normal range<br>The normal blood sugar range for adults and children above the age of 7 is between 3.9mmol/L (70 mg/dl) and 6.1 mmol/L (110mg/ dl).'	You can use the second test a few days or weeks later in order to confirm your result.	• There are some subtle differences in colour on the colour chart. (MR)

HR – high risk concern; MR – moderate risk concern; LR – low risk concern. FIT – faecal immunochemical test; FOB – faecal occult blood; FSH – follicle-stimulating hormone; HIV – human immunodeficiency virus; IFU – Instructions For Use; MAU – microalbuminuria; NHS – national health service; NS – not specified; PCR – polymerase chain reaction; PSA – prostate specific antigen; STI – sexually transmitted infection; TSH – thyroid stimulating hormone; UTI – urinary tract infection; WHO – World Health Organisation.

[1] NICE CKS. Bowel screening: What is the NHS bowel screening programme in the UK? UK: NICE CKS; 2024 [updated Apr 2024. Available from: https://cks.nice.org.uk/topics/bowel-screening/background-information/the-nhs-bowel-screening-programme/, accessed 08/03/2025].

[2] NICE CKS. Prostate cancer: How should I assess a person with suspected prostate cancer? UK: NICE CKS; 2022 [updated Sept 2024. Available from: https://cks.nice.org.uk/topics/prostate-cancer/diagnosis/assessment/, accessed 20/03/2025].

[3] NICE CKS. Coeliac disease: How should I assess a person with suspected coeliac disease? UK: NICE CKS; 2020 [updated May 2020. Available from: https://cks.nice.org.uk/topics/coeliac-disease/diagnosis/assessment/, accessed 11/03/2025].

[4] NICE CKS. Thyroid disease: assessment and management. NICE guideline [NG145]. Recommendations. UK: NICE CKS; 2019 [updated 12 Oct 2023. Available from: https://www.nice.org.uk/guidance/ng145/chapter/recommendations, accessed 11/03/2025].

[5] NICE CKS. Anaemia - iron deficiency: What investigations should I arrange to confirm iron deficiency anaemia? UK: NICE CKS; 2023 [updated Aug 2024. Available from: https://cks.nice.org.uk/topics/anaemia-iron-deficiency/diagnosis/investigations/, accessed 11/03/2025].

[6] The World Health Organization. WHO guideline on use of ferritin concentrations to assess iron status in individuals and populations. Geneva: WHO; 2020 [Available from: https://www.who.int/publications/i/item/9789240000124, accessed 18/02/2025].

[7] NICE CKS. Vitamin D deficiency in adults: When should I suspect or test for vitamin D deficiency? UK: NICE CKS; 2021 [updated Jan 2022. Available from: https://cks.nice.org.uk/topics/vitamin-d-deficiency-in-adults/diagnosis/diagnosis/, accessed 11/03/2025].
